# Supplementary material for: Does higher early neonatal mortality in boys reverse over the neonatal period? A pooled analysis from three trials of Nepal
Source: BMJ Open. 2022 May 18;12(5):e056112. doi: 10.1136/bmjopen-2021-056112 (PMC9121405; doi:10.1136/bmjopen-2021-056112)
Supplement: Supplementary data [file bmjopen-2021-056112supp001.pdf]

ANNEX

Table 1: Mortality Rates by age and sex for NNIPS-3

NNIPS-3 (1999-2000) N=4,127										
	Males (N=2082)					Females(N=2045)				
**Age Group**	**Deaths**	**Person Year**	**Death Rate**	**Probability of Dying**	**Cumulative Probability (Mortality)**	**Deaths**	**Person Year**	**Death Rate**	**Probability of Dying**	**Cumulative Probability (Mortality)**
0-1day	35	2064	0.0169575	.017	.0168	25	2031.5	0.0123063	.0123	.0122
1-3 days	24	4055	0.0059186	.0118	.0284	13	4014.0	0.0032387	.0065	.0186
3-7 days	8	8063	0.0009922	.004	.0322	16	7973.0	0.0020068	.008	.0265
7-14 days	13	14051	0.0009252	.0065	.0385	14	13852.0	0.0010107	.0071	.0333
14-21 days	10	13964	0.0007161	.005	.0433	8	13782.0	0.0005805	.0041	.0372
21-28 days	2	13927	0.0001436	.001	.0442	5	13744.0	0.0003638	.0025	.0397
**Total**	**92**	**56123**				**81**	**55396.48**			

Table 2: Mortality Rates by age and sex for Chlorhexidine Study

CHX Study(2002-2006) N=23,644										
	Males (N= 12,188)					Females(N=11,456)				
**Age Group**	**Deaths**	**Person Year**	**Death Rate**	**Probability of Dying**	**Cumulative Probability (Mortality)**	**Deaths**	**Person Year**	**Death Rate**	**Probability of Dying**	**Cumulative Probability (Mortality)**
0-1day	141	12065	0.0116866	.0117	.0116	112	11362	0.0098573	.0099	.0098
1-3 days	96	23924	0.0040127	.008	.0195	80	22559	0.0035463	.0071	.0168
3-7 days	57	47541	0.0011990	.0048	.0242	44	44879	0.0009804	.0039	.0207
7-14 days	39	82881	0.0004706	.0033	.0274	43	78208	0.0005498	.0038	.0244
14-21 days	26	82648	0.0003146	.0022	.0296	36	77929	0.0004620	.0032	.0276
21-28 days	12	82477	0.0001455	.001	.0305	23	77685	0.0002961	.0021	.0296
**Total**	**371**	**331536**				**338**	**312622**			

Table 3: Mortality Rates by age and sex for NOMS

NOMS (2010-2017) N=31,958										
	Males (N= 16,533)					Females(N=15,425)				
**Age Group**	**Deaths**	**Person Year**	**Death Rate**	**Probability of Dying**	**Cumulative Probability (Mortality)**	**Deaths**	**Person Year**	**Death Rate**	**Probability of Dying**	**Cumulative Probability. (Mortality)**
0-1day	225	16343	0.0137674	.0138	.0137	189	15266	0.0123804	.0124	.0123
1-3 days	133	32377	0.0041079	.0082	.0217	107	30273	0.0035345	.0071	.0193
3-7 days	87	64045	0.0013584	.0054	.027	60	59997	0.0010000	.004	.0232
7-14 days	44	110578	0.0003979	.0028	.0298	38	103879	0.0003658	.0026	.0257
14-21 days	47	109206	0.0004304	.003	.0327	33	102730	0.0003212	.0022	.0279
21-28 days	12	106394	0.0001128	.0008	.0334	22	100177	0.0002196	.0015	.0294
**Total**	**548**	**438943**				**449**	**412322**			

Table 4: Mortality Rates by age and sex for Pooled Study

Pooled Nepal Datasets (1999-2017) N=59,729										
	Males (N=30803)					Females(N=28926)				
**Age Group**	**Deaths**	**Person Year**	**Death Rate**	**Probability of Dying**	**Cumulative Probability (Mortality)**	**Deaths**	**Person Year**	**Death Rate**	**Probability of Dying**	**Cumulative Probability (Mortality)**
0-1day	401	30472	0.0131596	.0132	.0131	326	28660	0.0113748	.0114	.0113
1-3 days	253	60356	0.0041918	.0084	.0213	200	56846	0.0035183	.007	.0182
3-7 days	152	119649	0.0012704	.0051	.0263	120	112849	0.0010634	.0043	.0224
7-14 days	96	207511	0.0004626	.0032	.0294	95	195939	0.0004848	.0034	.0257
14-21 days	83	205818	0.0004033	.0028	.0322	77	194441	0.0003960	.0028	.0284
21-28 days	26	202798	0.0001282	.0009	.033	50	191606	0.0002610	.0018	.0302
**Total**	**1011**	**826603**				**868**	**780340**			
