# Supplementary material for: Does higher early neonatal mortality in boys reverse over the neonatal period? A pooled analysis from three trials of Nepal
Source: BMJ Open. 2022 May 18;12(5):e056112. doi: 10.1136/bmjopen-2021-056112 (PMC9121405; doi:10.1136/bmjopen-2021-056112)
Supplement: Author's manuscript [file bmjopen-2021-056112.draft_revisions.pdf]

BMJ Open is committed to open peer review. As part of this commitment we make the peer review history of every article we publish publicly available.

When an article is published we post the peer reviewers' comments and the authors' responses online. We also post the versions of the paper that were used during peer review. These are the versions that the peer review comments apply to.

The versions of the paper that follow are the versions that were submitted during the peer review process. They are not the versions of record or the final published versions. They should not be cited or distributed as the published version of this manuscript.

BMJ Open is an open access journal and the full, final, typeset and author-corrected version of record of the manuscript is available on our site with no access controls, subscription charges or pay-per-view fees (http://bmjopen.bmj.com).

If you have any questions on BMJ Open's open peer review process please email info.bmjopen@bmj.com

BMJ Open

Higher early neonatal mortality in boys is reversed in the 4th week of life: A pooled analysis from Nepal.

Journal:	*BMJ Open*
Manuscript ID	bmjopen-2021-056112
Article Type:	Original research
Date Submitted by the Author:	05-Aug-2021
Complete List of Authors:	Subedi, Seema; Johns Hopkins University Bloomberg School of Public Health, International Health<br>Katz, Joanne; Johns Hopkins University Bloomberg School of Public Health, International Health<br>Erchick, Daniel; Johns Hopkins University Bloomberg School of Public Health, International Health<br>Verhulst, Andrea; University of Pennsylvania, Population Studies Center<br>Khatry, Subarna; Nepal Nutr Intervent Project Sarlahi<br>Mullany, Luke C.; Johns Hopkins University Bloomberg School of Public Health, International Health<br>Tielsch, James; George Washington University School of Public Health and Health Services, Global Health<br>LeClerq, Steven; Johns Hopkins University Bloomberg School of Public Health, International Health; Nepal Nutr Intervent Project Sarlahi<br>Christian, P; Johns Hopkins University Bloomberg School of Public Health, International Health<br>West, KP; Johns Hopkins University Bloomberg School of Public Health, International Health<br>Guillot, Michel; University of Pennsylvania, Population Studies Center
Keywords:	EPIDEMIOLOGY, Community child health < PAEDIATRICS, NEONATOLOGY

SCHOLARONE™ Manuscripts

I, the Submitting Author has the right to grant and does grant on behalf of all authors of the Work (as defined in the below author licence), an exclusive licence and/or a non-exclusive licence for contributions from authors who are: i) UK Crown employees; ii) where BMJ has agreed a CC-BY licence shall apply, and/or iii) in accordance with the terms applicable for US Federal Government officers or employees acting as part of their official duties; on a worldwide, perpetual, irrevocable, royalty-free basis to BMJ Publishing Group Ltd ("BMJ") its licensees and where the relevant Journal is co-owned by BMJ to the co-owners of the Journal, to publish the Work in this journal and any other BMJ products and to exploit all rights, as set out in our licence.

The Submitting Author accepts and understands that any supply made under these terms is made by BMJ to the Submitting Author unless you are acting as an employee on behalf of your employer or a postgraduate student of an affiliated institution which is paying any applicable article publishing charge ("APC") for Open Access articles. Where the Submitting Author wishes to make the Work available on an Open Access basis (and intends to pay the relevant APC), the terms of reuse of such Open Access shall be governed by a Creative Commons licence – details of these licences and which Creative Commons licence will apply to this Work are set out in our licence referred to above.

Other than as permitted in any relevant BMJ Author's Self Archiving Policies, I confirm this Work has not been accepted for publication elsewhere, is not being considered for publication elsewhere and does not duplicate material already published. I confirm all authors consent to publication of this Work and authorise the granting of this licence.

For peer review only

Higher early neonatal mortality in boys is reversed in the 4th week of life: A pooled analysis from Nepal

Authors: Seema Subedi[1], Joanne Katz[1], Daniel J. Erchick[1], Andrea Verhulst[2], Subarna K. Khatry[3], Luke C. Mullany[1], James M. Tielsch[4], Steven C. LeClerq[1,3], Parul Christian[1], Keith P. West Jr.[1] , Michel Guillot[2]

Affiliations:

[1] Department of International Health, Johns Hopkins Bloomberg School of Public Health

[2] Department of Sociology & Population Studies Center, University of Pennsylvania

[3] Nepal Nutrition Intervention Project – Sarlahi, Kathmandu, Nepal.

[4] Department of Global Health, George Washington University Milken Institute School of Public Health.

Emails: ssubedi2@jhu.edu, jkatz1@jhu.edu, derchick@jhu.edu, verhulst@sas.upenn.edu, skhatry@wlink.com.np, lmullany@gmail.com, jtielsch@email.gwu.edu, sleclerq@mos.com.np, pchrist1@jhu.edu, west1@jhu.edu, miguillo@sas.upenn.edu

Correspondence: Seema Subedi, MPH. Department of International Health, Johns Hopkins Bloomberg School of Public Health, 615 N. Wolfe Street, Baltimore, MD, 21205, United States. ssubedi2@jhu.edu

Word Count:

Abstract: 299

Main Manuscript (including tables): 3726

For peer review only

ABSTRACT

Introduction:

In high-income settings, neonatal mortality is generally 20% higher in males than females, due to biological phenomena. This is not consistently observed in low- and middle-income settings. South Asian countries have reported a sex-related mortality reversal (females higher than males) in the late neonatal period. Only a few studies have examined more finely categorized age patterns of neonatal mortality by sex, especially in the first few days of life.

Methods:

We analyzed data from three community-based randomized controlled trials conducted in rural Nepal. Separately for each data source, and for the overall pooled dataset (n=59,729), we calculated mortality rates for males and females by ages (0-1, 1-3, 3-7, 7-14, 14-21 and 21-28 days) and estimated hazard ratios (HR) using Cox proportional hazard models for male versus female mortality.

Results:

Neonatal mortality was higher in males than females in individual studies and pooled analysis: 44.2 vs. 39.7 in males and females in 1999-2000; 30.0 vs. 29.6 in 2002-2005; 33.4 vs. 29.4 in 2010-2017; and 33.0 vs. 30.2 in the pooled analysis. Pooled data found that early neonatal mortality (HR=1.17; 95% CI: 1.06-1.30) was significantly higher in males than females. All individual datasets showed a reversal in mortality pattern by sex after the 3rd week of life. In the 4th week, a reversal was observed, with mortality in females 2.43 times higher than males (HR=0.41; 95% CI: 0.31-0.79).

Conclusions:

Males had higher mortality in the early (first week) neonatal period followed by no sex difference in weeks 2 and 3 and a reversal in risk in the 4th week of life, with females dying at more than twice the rate of males. This may be a result of gender discrimination and social norms in this setting. Interventions to reduce gender discrimination at the household level may reduce female neonatal mortality.

For peer review only - http://bmjopen.bmj.com/site/about/guidelines.xhtml

STRENGTHS AND LIMITATIONS OF THE STUDY

- Since the neonates were followed at frequent intervals, we could examine the sex differentials in neonatal mortality at more detailed age (0-1, 1-3, 3-7, 7-14, 14-21, and 21-28 days), which have not been seen in other studies.
- Since we used data from three different trials in the same settings, it was appropriate to analyze by pooling the data.
- We could not examine the determining factors for the main result of the study and our discussions are based on the existing literature.

INTRODUCTION

Since the 1960s, high-income countries (HICs) have reported higher neonatal mortality rates in males than females.[1-6] For overall neonatal mortality, males are at an approximately 20% greater risk of neonatal mortality than females. These differences are explained primarily as biologically driven phenomenon with the predominant causes of death being non-infectious.[2, 3, 5, 6] Several factors associated with higher neonatal mortality in males include intrauterine growth restriction, respiratory distress syndrome, prematurity and birth asphyxia.[7-10] Studies examining immunologic differences in animal models have showed that females have stronger innate and humoral responses to infection, making them better able to fight infection.[11-13] These studies also show that there is an association between sex hormones and immune function, where testosterone in males suppresses the immune system, while estradiol and progesterone in females improve both the innate and humoral immune responses.[11-13] Males also have higher birthweights than females, leading to a higher risk of complications during delivery and injuries at birth, although in general, low birthweight is associated with higher mortality.[2, 11, 14-19] Data from HICs show that the mortality in males is higher than females not only during the neonatal period, but also after the neonatal period, through infancy and beyond.[2, 20]

In low- and middle-income countries (LMICs) with higher neonatal mortality (more than 30 per 1,000 live births), sex differences in neonatal mortality have been inconsistent. A multi-country study in Sub-Saharan Africa reported higher neonatal mortality ratios for males to females ranging from 1.1. to 1.6.[21] Similarly, an Indonesian study of Demographic and Health Surveys (DHS) data reported an adjusted sex difference in neonatal mortality of 1.49 times higher in males.[22] However, a Pakistani study reported an overall sex difference of 0.82, indicating higher neonatal mortality risk in females.[23]

For peer review only - http://bmjopen.bmj.com/site/about/guidelines.xhtml

Separating neonatal mortality into early and late neonatal mortality, the literature from both HICs and LMICs generally show that males have higher rates of mortality than females in the early neonatal period (first week of life).[4, 5, 19, 23, 24] The extent of these differences varies by factors like level of neonatal mortality, causes of neonatal mortality, and other region-specific factors. However, sex differences in mortality during the late neonatal period have not been consistent.

Evidence from South Asia has suggested that although males are at higher risk of death in the early neonatal period, this pattern can reverse in the late neonatal period.[23, 24] A study by Rosenstock et al., which used one of the datasets of our analysis (Chlorhexidine Study), showed there was a reversal in the mortality pattern by sex in Nepal.[19] In the early neonatal period, males were at 20% higher mortality risk, assumed to be due to biological factors, whereas in the late neonatal period, girls were at a 43% higher mortality risk. This was associated with ethnicity and the gender structure of siblings in the family rather than by gender preference alone, where girls born to families with only girls had higher risk.[19] In an urban Pakistani study, where overall neonatal mortality was lower in males (0.82), the sex differences in early and late neonatal mortality were 1.21 and 0.28 respectively, indicating a reversal of risk in the later weeks of the neonatal period.[23] Differential health care-seeking behaviors and gender preference for male infants, have been reported as explanations for higher late neonatal and infant mortality in females.[23-28]

Some South Asian studies have examined sex differences in post-neonatal mortality. An analysis of data from a randomized trial in rural northern India comparing sex differences in mortality during the neonatal period and beyond showed that males had 1.25 times higher neonatal mortality in the 1st week of life. In the post-neonatal period, however, females had significantly higher mortality; 1.4 and 1.7 times higher in days 29-180 and days 181-365, respectively.[15] Factors associated with excess female mortality in the post-neonatal period were caste and mother's occupation (higher for mothers working outside the home).

A recent study with 297,509 live births in India and Pakistan showed that both overall and early neonatal mortality risk were significantly higher in males than females. However, there was no significant difference by sex in late neonatal mortality, and mortality between 29-42 days.[7]

For peer review only - http://bmjopen.bmj.com/site/about/guidelines.xhtml

Given that some South Asian countries showed a reversal in neonatal mortality, and others have not, we examined data from three, sequential, community-based randomized controlled trials (RCTs) conducted in the District of Sarlahi located in the east-central, southern rural plains (Terai) of Nepal. These studies included frequent in-person follow-up of all live born infants with exact date of deaths, allowing us to analyze sex differences in mortality by more finely categorized ages (0-1 day, 1-3 days, 3-7 days, 7-14 days, 14-21 days, and 21-28 days). Examining the sex differential in mortality by more detailed ages can help us pinpoint the age at which the pattern of sex difference in mortality changes or reverses, which could help us plan interventions accordingly.

For peer review only

For peer review only - http://bmjopen.bmj.com/site/about/guidelines.xhtml

METHODS

Characteristics of the datasets used in this analysis are provided in Table 1. The studies provide pregnancy cohorts from 1999 through 2017. Child follow-up duration ranged from 28 days to 5 years. All studies were community-based RCTs conducted in the same rural community of Nepal by the Nepal Nutrition Intervention Project, Sarlahi (NNIPS). The first study, NNIPS-3, followed pregnancies and births in the study area from 1999-2000, to look at the effect of antenatal multiple micronutrient supplementation on birth outcomes and the health of their children (Clinicaltrials.gov (NCT00115271)). [29] The second, the Chlorhexidine Study (CHX), followed participants from 2002-2006 to assess if a chlorhexidine body wipe and/or chlorhexidine application to the umbilical stump reduced neonatal mortality (Clinicaltrials.gov (NCT00109616)).[30] The third, the Nepal Oil Massage Study (NOMS), followed participants from 2010-2017 to evaluate the impact of sunflower versus mustard seed oil massage on neonatal mortality (Clinicaltrials.gov (NCT01177111)). In each study, vital status of newborns at birth and through 28 days of life was ascertained in a prospective follow-up done by study teams and date of death was recorded. In the NNIPS-3 and CHX studies, live vs stillbirths were self-reported by mothers. In NOMS, infants were considered born alive if the baby moved, cried or breath after the birth.

Individual-level data for each pregnancy and live birth included date and type (live/stillbirth) of outcome, date of death, length of follow-up, sex of the infant, and whether the birth was a singleton or multiple. Only live births were used for this analysis. For each individual dataset, we calculated survival times for live births using dates of birth and death. Survival times were split into age categories (0-1 (i.e. first 24 hours), 1-3, 3-7, 7-14, 14-21, and 21-28 days). The total deaths and person-time in each category were used to calculate death rates, and probability of dying with 95% CI in those groups, separately for males and females. Differences in the probability of dying between males and females were visualized using mortality curves. Cox regression was used to estimate hazard ratios with 95% CI for male versus female mortality for overall neonatal mortality (0-28 days), early neonatal mortality (0-7 days), late neonatal mortality (7-28 days), and for the more finely categorized age groups described above. Datasets were then pooled to conduct the same analyses.

The research proposal (IRB Protocol number 827014, and IRB number 8) was considered exempt by the Institutional Review Board at the University of Pennsylvania , authorized by 45 CFR 46.101, category 4. NNIPS-3, CHX and NOMS studies were approved by the institutional review board

For peer review only

(IRB) of the Johns Hopkins Bloomberg School of Public Health. NNIPS-3 and CHX were approved by the IRB of the Institute of Medicine, Tribhuvan University in Nepal. NOMS was approved by the Nepal Health Research Council in Nepal. Verbal consent was obtained from women for their participation and their infants, for all studies.

Patients or the public were not involved in the design, conduct, reporting, or dissemination plans for this research.

For peer review only

For peer review only - http://bmjopen.bmj.com/site/about/guidelines.xhtml

Table 1. Methodologies of studies included in the analysis of sex specific mortality by age.

Study	Study Design	Birth Cohorts	Total FUP time	Neonatal FUP visits	Neonatal-level Intervention	Total LB in analysis	Number of Neonatal Deaths	Cumulative Neonatal Mortality (per 1000 LBs)
**NNIPS-3**	Randomized Controlled Community Trial	1999-2000	28 days *	Day 1, 2, 3, 4, 5, 6, 7, 8, 9, 10, 17, 24 and 31.	None	M=2,082	M= 92	M=44.2
						F= 2,045	F=81	F=39.7
						**Total= 4,127**	**Total=173**	**Overall=41.9**
**CHX Study**	Randomized Controlled Community Trial	2002-2006	28 days	Day 1, 2, 3, 4, 6, 8, 10, 12, 14, 21 and 28.	Chlorhexidine and Placebo wipe	M= 12,188	M=371	M=30.5
						F= 11,456	F=338	F=29.6
						**Total=23,644**	**Total=709**	**Overall=30.0**
**NOMS**	Randomized Controlled Community Trial	2010-2017	28 days	Day 1, 3, 7, 10, 14, 21 and 28.	Sunflower Oil and Mustard Oil massage	M=16,533	M=548	M=33.4
						F=15,425	F=449	F=29.4
						**T= 31,958**	**T=997**	**Overall=31.4**
**Total**						**59,729**	**1,879**	**31.6**

**In NNIPS-3 follow-up went beyond 28 days, but for the other 2 studies, it went through 28 days only.*

For peer review only

RESULTS

The overall neonatal mortality risk was higher in males than females in the individual studies as well as the pooled analysis (N=59,729 live births) (Table 1). Neonatal mortality was 41.9 per 1,000 live births (LB) (44.2 versus 39.7 in males and females), 30.0 per 1,000 LB (30.5 versus 29.6 in males and females), 31.4 per 1,000 LB (33.4 versus 29.4 in males and females) and 31.6 per 1000 LB (33.0 versus 30.2 in males and females) in 1999, 2002, 2010 and the pooled analysis respectively. Child's sex was missing for a very small number of neonatal deaths (1/174 in NNIPS-3, none in CHX study, and 4/1001 in NOMS).

The 1999 NNIPS-3 study found that more males than females died early (0-1 day, 1-3 days), then the rates for males and females converged, until a reversal was seen in the 4th week of life (Figure 1), (HR= 0.39; 95% CI: 0.08-2.03). The 2002 Chlorhexidine study found males had a higher mortality than females in the early neonatal period (0-1 day, 1-3 days and 3-7 days), then mortality quickly reversed after the first week and continued until the 4th week (Figure 1), (HR= 0.49; 95%CI: 0.24-0.98). The 2010 NOMS study had higher mortality in males than females in the early neonatal period (0-1 day, 1-3 days and 3-7 days), then mortality converged in the 2nd and 3rd weeks, followed by a reversal after the 3rd week (Figure 1), (HR= 0.51; 95% CI: 0.25-1.04) . A common finding in all three studies was that there was a reversal after the 3rd week of life, where female mortality was higher than for males, although this reversal was statistically significant only in the CHX study (Table 2). Our pooled analysis showed mortality among males was higher until the 2nd week (0-1 day, 1-3 days, 3-7 days and 7-14 days), followed by similar rates during the 3rd week (14-21 days), followed by a statistically significant reversal in the 4th week of life (21-28 days) (Figure 2). For the pooled analysis, results from Cox regression showed that early neonatal mortality (HR=1.17; 95% CI: 1.06-1.30) was significantly higher in males than females, and the 4th week mortality reversed with 2.43 (95% CI: 1.26-3.33) times higher in females than males (HR= 0.41; 95% CI: 0.31-0.79) (Table 3).

For peer review only

Table 2: Hazard Ratio for Neonatal Mortality for Individual Studies

	NNIPS-3 (1999-2000) N=4127			CHX Study (2002-2006) N=23,644			NOMS (2010-2017) N=31,958		
**Age Category**	**Hazard Ratio (M/F)**	**95%CI**	**p-value**	**Hazard Ratio (M/F)**	**95%CI**	**p-value**	**Hazard Ratio (M/F)**	**95%CI**	**p-value**
Overall Neonatal (0-28days)	**1.11**	0.83, 1.51	0.458	**1.03**	0.89, 1.20	0.652	**1.14**	1.01, 1.29	0.037
Early Neonatal (0-7 days)	**1.22**	0.85, 1.75	0.272	**1.17**	0.99, 1.40	0.067	**1.16**	1.02, 1.34	0.029
Late Neonatal (7-28 days)	**0.91**	0.53, 1.57	0.744	**0.71**	0.53, 0.96	0.024	**1.04**	0.79, 1.38	0.777
**More Finely Categorized Ages**									
0-1 day	**1.37**	0.82, 2.30	0.224	**1.18**	0.92, 1.52	0.18	**1.11**	0.92, 1.35	0.284
1-3 days	**1.82**	0.93, 3.58	0.081	**1.13**	0.84, 1.52	0.415	**1.16**	0.90, 1.50	0.247
3-7 days	**0.49**	0.21, 1.16	0.105	**1.22**	0.82, 1.81	0.316	**1.35**	0.98, 1.89	0.068
7-14 days	**0.91**	0.43, .95	0.819	**0.85**	0.55, 1.32	0.482	**1.08**	0.70, 1.68	0.705
14-21 days	**1.23**	0.49, 3.12	0.658	**0.68**	0.41, 1.13	0.135	**1.33**	0.86, 2.10	0.198
21-28 days	**0.39**	0.08, 2.03	0.267	**0.49**	0 .24, 0.98	0.046	**0.51**	0.25, 1.04	0.063

NNIPS – Nepal Nutrition Intervention Project Sarlahi, CHX – Chlorhexidine intervention trial, NOMS – Nepal Oil Massage Study

For peer review only

Table 3: Probability of Dying and Hazard Ratio of Neonatal Mortality for Pooled Analysis

POOLED (1999-2017) N=59,729

Age Category	Males(N=30,803)			Females (N=28,926)			Hazard Ratio (M/F)	95%CI	p-value
	Deaths	Person Year	Probability of Dying	Deaths	Person Year	Probability of Dying			
Overall Neonatal (0-28days)	1011	826,603	.0086	868	780,341	.0078	1.09	1.00, 1.20	0.045
Early Neonatal (0-7 days)	806	210,476	.0268	646	198,355	.0228	1.17	1.06, 1.30	0.002
Late Neonatal (7-28 days)	205	616,127	.0023	222	581,986	.0027	0.87	0.72, 1.05	0.158
**More Finely Categorized Ages**									
0-1 day	401	30,472	.0132	326	28,660	.0114	1.15	1.00, 1.34	0.051
1-3 days	253	60,356	.0084	200	56,846	.007	1.19	0.99, 1.43	0.064
3-7 days	152	119,649	.0051	120	112,849	.0043	1.19	0.94, 1.52	0.146
7-14 days	96	207,511	.0032	95	195,939	.0034	0.94	0.72, 1.27	0.745
14-21 days	83	205,818	.0028	77	194,441	.0028	1.01	0.75, 1.39	0.909
21-28 days	26	202,798	.0009	50	191,606	.0018	0.41	0.31, 0.79	0.003

For peer review only

DISCUSSION

Our study found higher mortality in males than females early in the neonatal period followed by a reversal in the 4th week of life in each of the individual studies. This work extends that of Rosenstock et al. to include a longer time span from 1999 through 2017 in the same geographic area.[19] In the pooled analysis, this reversal was statistically significant and the mortality hazard ratio was 2.43 times higher in females than males in the 4th week of life. This is similar to the findings by Rosenstock et al. in rural Nepal and Jehan et al. in urban Pakistan although they compared only early and late neonatal mortality and found a reversal in the late neonatal period.[19, 23] However, a recent study in India and Pakistan showed higher male mortality in the early neonatal period, but no significant difference in male and female mortality in the late neonatal period or between 29-42 days.[7] In that study, the first follow-up was within 48 hours of delivery, with one more visit at 42-day post-partum.[7] Our analysis had more frequent visits and a prospective record of exact date of death.

Another large randomized controlled trial rural North India, which examined differences in the post-neonatal period, found a reversal in 29-180 and 181-365 days after birth.[15] They followed live births on day 29 after the infant's birth and at ages 3, 6, 9 and 12 months to obtain vital status of the infant.[15] For this reason, they analyzed the difference in the post-neonatal period but could not do so in the late neonatal period. Our study with rigorous follow-up within the neonatal period allowed us to examine the sex differences in more finely categorized ages and identify that the reversal took place as early as the 4th week of life.

Our previous work with the 2002-2005 dataset further examined possible reasons for sex differences in mortality, finding that ethnicity, differential neonatal care seeking behavior and prior family composition with multiple daughters were important factors associated with higher late neonatal mortality in females.[19, 31] In Nepal, gender discrimination originates at the household level. Since the 1980s, when the World Fertility Surveys first documented evidence of son preference, Nepal has been categorized as a country with a high level of this preference.[32] This practice is still common, as seen in a 2012 survey on 1,000 Nepalese men aged 18-49 showing that the majority (90%) believed that a man with only daughters is unfortunate and not having a son reflects a lack of moral virtue. Nearly half said that a woman's important roles are limited to taking care of her home and cooking for her family. Married women reported that maintaining an income-generating job is precluded by care-giving for small children

For peer review only - http://bmjopen.bmj.com/site/about/guidelines.xhtml

(32%), lack of permission from other household/family decision makers (19%), and the workload at home (18%). Only 26% of married Nepalese women reported making independent decisions regarding their own health care.[33] Many women are still restricted to the private sphere with unpaid work. Women work for an estimated average of 268 minutes a day on household chores, whereas men work only 56 minutes.[34] This deprives women of quality education, awareness and exposure. Socially, sons are given preference because of the various cultural and economic roles that are believed to be performed by sons only: performing lighting of the funeral pyre, continuing the family lineage and providing old age economic security for their parents, whereas girls are considered an economic liability because they have to live in their parents' home until marriage, for which a dowry must be provided to the groom's family. Upon marriage they become part of the economy of the husband's family, hence being an economic drain on the family from birth onwards.[35] Nepal's patrilineal and patrilocal social structure combined with socio-economic and religious values leads to son preference and gender discrimination.

A systematic review found higher care seeking behavior for male than female neonates in seventeen studies in South Asia, particularly in households with older female siblings.[36] In addition, for male babies, care-seeking was more frequent, from better qualified care providers, and with higher expenditure compared to females. Studies also have consistently shown that households with female children were more likely to report discrimination, because family members perceived that care for illness was not so important, leading to reduced care-seeking.[36] Similar to Nepal, a 2011 UNICEF report on China also indicated that the discrimination against female infants was highest for those who had older female siblings.[37]

In Nepal, where most children are exclusively breastfed, the median duration of breastfeeding for males and females are 4.2 months and 4.1 months. [38] Hence this is an unlikely explanation for the reversal of mortality in the 4th week of life. The differential may be explained by poorer nutrition, care and rest provided to mothers giving birth to daughters, son preference being the root cause of discrimination in the family. It could also point to specific parental behaviors that takes place starting at that age and suggests a critical age window for intervention. However, the 3-week threshold could also just be an indication that the gender discrimination starts from birth or even earlier, but takes at least three weeks for the biological and natural survival

For peer review only - http://bmjopen.bmj.com/site/about/guidelines.xhtml

advantage of females to be overcome by the social advantage of males. Further studies could explore more about why this reversal starts specifically in the 4th week of life.

Neonatal mortality in Nepal has continued to decrease from 39 to 21 per 1,000 live births, with male neonatal mortality decreasing from 52 to 24 per 1000 LB (reduction of 28%) and female neonatal mortality decreasing from 43 to 17 per 1000 LB (reduction of 26%) from 2001 to 2016.[33, 38] Given that males have a biological disadvantage in neonatal survival, one could have expected female neonatal mortality to have decreased more than for males. If female mortality could decrease more than it has, this could contribute to a greater reduction in overall neonatal mortality. Although neonatal mortality in Nepal was decreasing from 2001 to 2016, it still contributed to a higher percentage of under-5 child mortality because mortality among older children has decreased faster than neonatal mortality.[39] If this trend continues, the Sustainable Development Goals (SDG) target of reducing neonatal mortality to 12 per 1,000 live births by 2030 in Nepal will be difficult to achieve.[40] Therefore, a focus on reducing female neonatal mortality could help meet the SDG for neonatal mortality and for gender equity. A cross national study from 138 countries also showed evidence that the Gender Inequality Index (GII) was positively associated with neonatal mortality.[41] Applying interventions to address gender discrimination by addressing cultural and social barriers at the household level may help reduce neonatal mortality.

In the early neonatal period, preterm birth is one of the main causes of deaths, while in the late neonatal period, sepsis and pneumonia account for more deaths.[42, 43] Studies also show that preterm birth is higher in males than females.[44-46] Given the biological susceptibility of males towards more early neonatal deaths both in the LMICs and HICs, it is not as easy to intervene. However, the main causes of late neonatal mortality like sepsis and pneumonia can be intervened on through improved care-seeking practices. So, if male and female children are provided with similar care-seeking practices in the late neonatal period, the sex-differences in neonatal mortality might be reduced, as in HICs.

Gender discrimination not only affects the quality of life of girls and women, but also reduces their survival in the neonatal period. Interventions to strengthen gender equality, such as counselling to woman and their family during antenatal care and postnatal care visits may be helpful to improve female and male neonatal survival. Since the reversal takes place during 4th

For peer review only - http://bmjopen.bmj.com/site/about/guidelines.xhtml

week of life, specific counselling interventions to parents and family could be targeted in the first 3 weeks of child's life.

The strength of this study is that it incorporates data from sequential randomized community trials from the same site sharing many similar field procedures carried out by the same, highly trained field teams over a 15-year period, so that pooling is reasonable and allows for a more precise analysis of sex differences in neonatal mortality. In addition, these studies have enrollment from pregnancy, which reduces the likelihood that early child deaths have been missed. Neonatal deaths have been tracked at frequent intervals to improve accuracy of age at death, enabling us to conduct survival analysis and Cox regression to obtain improved mortality estimates and hazard ratios.

This study was not able to examine the specific reasons for the mortality reversal. However, we have discussed possible reasons based on the existing literature. Further studies could examine why this reversal takes place as early as the 4th week of life and whether this reversal persists beyond the neonatal period.

CONCLUSION

Male mortality is higher than female in the early neonatal period, a biological phenomenon seen worldwide. However, this natural pattern is quickly reversed after the 3rd week of life in Nepal. This is likely due to gender discrimination and social norms that operate at household level. Implementing interventions to reduce gender discrimination at the household level could prevent this reversal and decrease female neonatal mortality, thereby reducing overall neonatal mortality and improving gender equity.

OTHER INFORMATION

Ethics approval

The research proposal (IRB Protocol number 827014, and IRB number 8) was considered exempt by the Institutional Review Board at the University of Pennsylvania , authorized by 45 CFR 46.101, category 4.

For peer review only - http://bmjopen.bmj.com/site/about/guidelines.xhtml

Consent for publication:

Not applicable.

Data Sharing:

No additional data available.

Competing interests:

The authors declare that they have no competing interests.

Funding source:

This work was supported by the National Institute for Child Health and Human Development (NICHD 1R01HD090082-01 and R01HD092411). NNIPS-3 was supported by Cooperative agreement HRN-A-00-97-00015-00 between Office of Health and Nutrition, US Agency for International Development (USAID), Washington, DC with additional support from the UNICEF Country Office, Kathmandu, Nepal (Grant number NA), and grant GH614 from the Bill & Melinda Gates Foundation (Seattle, WA). The CHX study was supported by R01HD044004 and R01HD038753 from the National Institutes of Health (Bethesda, MD); grant 810–2054 from the Bill and Melinda Gates Foundation (Seattle, WA) and Cooperative Agreements HRN-A-00-97-00015-00 and GHS-A-00-03-000019-00 between Johns Hopkins University and the Office of Health and Nutrition, US Agency for International Development (Washington, DC.). NOMS was supported by the National Institutes for Child Health and Development (HD060712) and the Bill & Melinda Gates Foundation (OPP1084399). The funders did not have a role in the design of the study, the data collection, nor the analysis, interpretation and writing of the manuscript.

Authors' contribution:

SS, JK, DE, AV, MG conceptualized and designed the study. SS conducted the analysis and wrote the manuscript. All authors reviewed results, discussed interpretations, and contributed to development and revision of the manuscript.

Acknowledgements:

We thank the women, infants, and their families who participated in this study, and the NNIPS project staff in Nepal. We are also grateful to Mrs. Lee S-F Wu from Johns Hopkins Bloomberg School of Public health for her contribution to the NNIPS-3 study.

For peer review only

For peer review only - http://bmjopen.bmj.com/site/about/guidelines.xhtml

REFERENCES

1 Elsmén E, Steen M, Hellström-Westas L. Sex and gender differences in newborn infants: why are boys at increased risk?. *Journal of Men's Health and Gender* 2004;1:303-11.

2 Drevenstedt GL, Crimmins EM, Vasunilashorn S, et al. The rise and fall of excess male infant mortality. *Proceedings of the National Academy of Sciences* 2008;105:5016-21.

3 Nathanson CA. Sex differences in mortality. *Annual review of sociology* 1984;10:191-213.

4 Hammoud EI. Studies in fetal and infant mortality. II. Differentials in mortality by sex and race. *American Journal of Public Health and the Nations Health* 1965;55:1152-63.

5 Waldron I. Patterns and causes of excess female mortality among children in developing countries. *World health statistics quarterly 1987; 40 (3): 194-210* 1987.

6 Gove WR, Carpenter GR. The fundamental connection between nature and nurture: a review of the evidence: Free Press 1982.

7 Aghai ZH, Goudar SS, Patel A, et al. Gender variations in neonatal and early infant mortality in India and Pakistan: a secondary analysis from the Global Network Maternal Newborn Health Registry. *Reproductive Health* 2020;17:1-11.

8 Hou L, Wang X, Li G, et al. Cross sectional study in China: fetal gender has adverse perinatal outcomes in mainland China. *BMC pregnancy and childbirth* 2014;14:372.

9 Aibar L, Puertas A, Valverde M, et al. Fetal sex and perinatal outcomes. *J Perinat Med* 2012;40:271-6.

10 Simchen MJ, Weisz B, Zilberberg E, et al. Male disadvantage for neonatal complications of term infants, especially in small-for-gestational age neonates. *The Journal of Maternal-Fetal & Neonatal Medicine* 2014;27:839-43.

11 Klein SL. Hormones and mating system affect sex and species differences in immune function among vertebrates. *Behav Processes* 2000;51:149-66.

12 Klein SL, Marson AL, Scott AL, et al. Neonatal sex steroids affect responses to Seoul virus infection in male but not female Norway rats. *Brain Behav Immun* 2002;16:736-46.

13 Klein SL. Hormonal and immunological mechanisms mediating sex differences in parasite infection. *Parasite Immunol* 2004;26:247-64.

14 Mishra SK, Ram B, Singh A, et al. Birth order, stage of infancy and infant mortality in India. *J Biosoc Sci* 2018;50:604-25.

15 Chowdhury R, Taneja S, Mazumder S, et al. Gender differences in infant survival: a secondary data analysis in rural North India. *BMJ open* 2017;7.

For peer review only - http://bmjopen.bmj.com/site/about/guidelines.xhtml

16 Yorita KL, Holman RC, Sejvar JJ, et al. Infectious disease hospitalizations among infants in the United States. *Pediatrics* 2008;121:244-52.

17 Elsmén E, Pupp IH, Hellström-Westas L. Preterm male infants need more initial respiratory and circulatory support than female infants. *Acta Paediatrica* 2004;93:529-33.

18 Baqui AH, Darmstadt GL, Williams EK, et al. Rates, timing and causes of neonatal deaths in rural India: implications for neonatal health programmes. *Bull World Health Organ* 2006;84:706-13.

19 Rosenstock S, Katz J, Mullany LC, et al. Sex differences in neonatal mortality in Sarlahi, Nepal: the role of biology and environment. *J Epidemiol Community Health* 2013;67:986-91.

20 Zhao D, Zou L, Lei X, et al. Gender differences in infant mortality and neonatal morbidity in mixed-gender twins. *Scientific reports* 2017;7:1-6.

21 Garenne M. Sex differences in health indicators among children in African DHS surveys. *J Biosoc Sci* 2003;35:601-14.

22 Titaley CR, Dibley MJ, Agho K, et al. Determinants of neonatal mortality in Indonesia. *BMC Public Health* 2008;8:232.

23 Jehan I, Harris H, Salat S, et al. Neonatal mortality, risk factors and causes: a prospective population-based cohort study in urban Pakistan. *Bull World Health Organ* 2009;87:130-8.

24 Katz J, West Jr KP, Khatry SK, et al. Risk factors for early infant mortality in Sarlahi district, Nepal. *Bull World Health Organ* 2003;81:717-25.

25 Nielsen BB, Liljestrand J, Hedegaard M, et al. Reproductive pattern, perinatal mortality, and sex preference in rural Tamil Nadu, south India: community based, cross sectional study. *BMJ* 1997;314:1521.

26 Willis JR. Perceived neonatal morbidities and healthcare utilization for neonatal health in rural Uttar Pradesh, India: effects of a behavior-change communication intervention: ProQuest 2007.

27 Pandey A, Sengupta PG, Mondal SK, et al. Gender differences in healthcare-seeking during common illnesses in a rural community of West Bengal, India. *Journal of Health, Population and Nutrition* 2002:306-11.

28 Howlader AA, Bhuiyan MU. Mothers' health-seeking behaviour and infant and child mortality in Bangladesh. *Asia-Pacific Population Journal* 1999;14:59.

29 Christian P, West KP, Khatry SK, et al. Effects of maternal micronutrient supplementation on fetal loss and infant mortality: a cluster-randomized trial in Nepal. *Am J Clin Nutr* 2003;78:1194-202.

For peer review only

For peer review only - http://bmjopen.bmj.com/site/about/guidelines.xhtml

30 Tielsch JM, Darmstadt GL, Mullany LC, et al. Impact of newborn skin-cleansing with chlorhexidine on neonatal mortality in southern Nepal: a community-based, cluster-randomized trial. *Pediatrics* 2007;119:e330-40.

31 Rosenstock S, Katz J, Mullany LC, et al. Sex differences in morbidity and care-seeking during the neonatal period in rural southern Nepal. *Journal of Health, Population and Nutrition* 2015;33:1-10.

32 Cleland, J, Verall, J., and Vasessen, M. Sex of Children and their Influence on Reproductive Behavior. World Fertility Survey Comparative Studies 1983.

33 Ministry of Health - MOH/Nepal, New ERA/Nepal, ICF. Nepal Demographic and Health Survey 2016 2017.

34 Budlender D. Moussié R. Making Care Visible Women's Unpaid Care Work in Nepal, Nigeria, Uganda and Kenya 2013.

35 Nanda P, Gautam A, Verma R, et al. Study on gender, masculinity and son preference in Nepal and Vietnam: International Center for Research on Women (ICRW) New Dehli 2012.

36 Ismail SA, McCullough A, Guo S, et al. Gender-related differences in care-seeking behaviour for newborns: a systematic review of the evidence in South Asia. *BMJ global health* 2019;4:e001309.

37 UNICEF. Boys and girls in the life cycle. 2011.

38 Family Health Division of the Department of Health Services of the Ministry of Health/Nepal, New ERA/Nepal, Macro O. Nepal Demographic and Health Survey 2001 2002.

39 Paudel, D., A. Thapa, P. R. Shedain, and B. Paudel. Trends and determinants of neonatal mortality in Nepal: Further analysis of the Nepal Demographic and Health Surveys, 2001-2011. 2013.

40 National Planning Commission. Sustainable Development Goals 2016–2030: National (Preliminary) Report. *Kathmandu: National Planning Commission* 2015.

41 Brinda EM, Rajkumar AP, Enemark U. Association between gender inequality index and child mortality rates: a cross-national study of 138 countries. *BMC Public Health* 2015;15:97-3 doi:10.1186/s12889-015-1449-3 [doi].

42 Dandona R, Kumar GA, Bhattacharya D, et al. Distinct mortality patterns at 0–2 days versus the remaining neonatal period: results from population-based assessment in the Indian state of Bihar. *BMC medicine* 2019;17:1-19.

For peer review only - http://bmjopen.bmj.com/site/about/guidelines.xhtml

43 Sankar MJ, Natarajan CK, Das RR, et al. When do newborns die? A systematic review of timing of overall and cause-specific neonatal deaths in developing countries. *Journal of perinatology* 2016;36:S1-S11.
44 Peelen MJ, Kazemier BM, Ravelli AC, et al. Impact of fetal gender on the risk of preterm birth, a national cohort study. *Acta Obstet Gynecol Scand* 2016;95:1034-41.
45 Zeitlin J, Ancel P, Larroque B, et al. Fetal sex and indicated very preterm birth: Results of the EPIPAGE study. *Obstet Gynecol* 2004;190:1322-5
46 Lao TT, Sahota DS, Suen SS, et al. The impact of fetal gender on preterm birth in a southern Chinese population. *The Journal of Maternal-Fetal & Neonatal Medicine* 2011;24:1440-3.

For peer review only

For peer review only - http://bmjopen.bmj.com/site/about/guidelines.xhtml

Figure 1: Sex difference in probability of dying for individual studies- NNIPS-3, CHX and NOMS (from left to right)

Figure 2: Sex difference in probability of dying for Pooled Analysis, Nepal 1999-2017

For peer review only

For peer review only - http://bmjopen.bmj.com/site/about/guidelines.xhtml

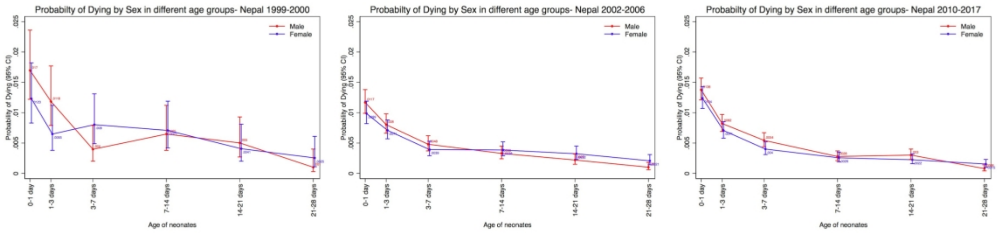

Figure 1: Sex difference in probability of dying for individual studies- NNIPS-3, CHX and NOMS (from left to right)

115x83mm (600 x 600 DPI)

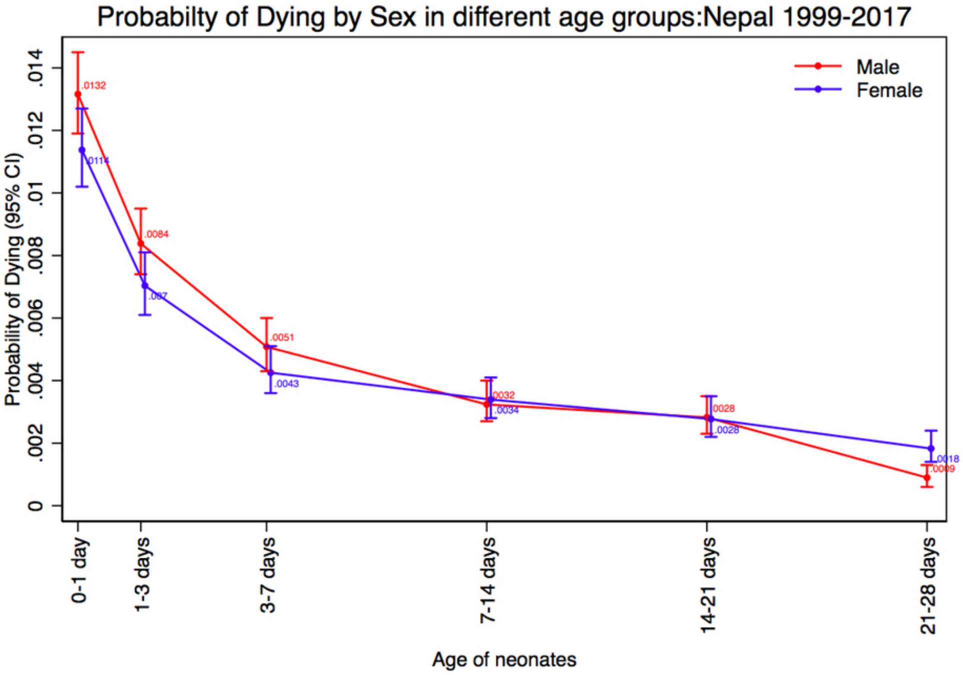

40x29mm (600 x 600 DPI)

ANNEX

Table 1: Mortality Rates by age and sex for NNIPS-3

NNIPS-3 (1999-2000) N=4,127										
	Males (N=2082)					Females(N=2045)				
**Age Group**	**Deaths**	**Person Year**	**Death Rate**	**Probability of Dying**	**Cumulative Probability (Mortality)**	**Deaths**	**Person Year**	**Death Rate**	**Probability of Dying**	**Cumulative Probability (Mortality)**
0-1day	35	2064	0.0169575	.017	.0168	25	2031.5	0.0123063	.0123	.0122
1-3 days	24	4055	0.0059186	.0118	.0284	13	4014.0	0.0032387	.0065	.0186
3-7 days	8	8063	0.0009922	.004	.0322	16	7973.0	0.0020068	.008	.0265
7-14 days	13	14051	0.0009252	.0065	.0385	14	13852.0	0.0010107	.0071	.0333
14-21 days	10	13964	0.0007161	.005	.0433	8	13782.0	0.0005805	.0041	.0372
21-28 days	2	13927	0.0001436	.001	.0442	5	13744.0	0.0003638	.0025	.0397
**Total**	**92**	**56123**				**81**	**55396.48**			

Table 2: Mortality Rates by age and sex for Chlorhexidine Study

CHX Study(2002-2006) N=23,644										
	Males (N= 12,188)					Females(N=11,456)				
**Age Group**	**Deaths**	**Person Year**	**Death Rate**	**Probability of Dying**	**Cumulative Probability (Mortality)**	**Deaths**	**Person Year**	**Death Rate**	**Probability of Dying**	**Cumulative Probability (Mortality)**
0-1day	141	12065	0.0116866	.0117	.0116	112	11362	0.0098573	.0099	.0098
1-3 days	96	23924	0.0040127	.008	.0195	80	22559	0.0035463	.0071	.0168
3-7 days	57	47541	0.0011990	.0048	.0242	44	44879	0.0009804	.0039	.0207
7-14 days	39	82881	0.0004706	.0033	.0274	43	78208	0.0005498	.0038	.0244
14-21 days	26	82648	0.0003146	.0022	.0296	36	77929	0.0004620	.0032	.0276
21-28 days	12	82477	0.0001455	.001	.0305	23	77685	0.0002961	.0021	.0296
**Total**	**371**	**331536**				**338**	**312622**			

For peer review only

Table 3: Mortality Rates by age and sex for NOMS

NOMS (2010-2017) N=31,958										
	Males (N= 16,533)					Females(N=15,425)				
**Age Group**	**Deaths**	**Person Year**	**Death Rate**	**Probability of Dying**	**Cumulative Probability (Mortality)**	**Deaths**	**Person Year**	**Death Rate**	**Probability of Dying**	**Cumulative Probability. (Mortality)**
0-1day	225	16343	0.0137674	.0138	.0137	189	15266	0.0123804	.0124	.0123
1-3 days	133	32377	0.0041079	.0082	.0217	107	30273	0.0035345	.0071	.0193
3-7 days	87	64045	0.0013584	.0054	.027	60	59997	0.0010000	.004	.0232
7-14 days	44	110578	0.0003979	.0028	.0298	38	103879	0.0003658	.0026	.0257
14-21 days	47	109206	0.0004304	.003	.0327	33	102730	0.0003212	.0022	.0279
21-28 days	12	106394	0.0001128	.0008	.0334	22	100177	0.0002196	.0015	.0294
**Total**	**548**	**438943**				**449**	**412322**			

Table 4: Mortality Rates by age and sex for Pooled Study

Pooled Nepal Datasets (1999-2017) N=59,729										
	Males (N=30803)					Females(N=28926)				
**Age Group**	**Deaths**	**Person Year**	**Death Rate**	**Probability of Dying**	**Cumulative Probability (Mortality)**	**Deaths**	**Person Year**	**Death Rate**	**Probability of Dying**	**Cumulative Probability (Mortality)**
0-1day	401	30472	0.0131596	.0132	.0131	326	28660	0.0113748	.0114	.0113
1-3 days	253	60356	0.0041918	.0084	.0213	200	56846	0.0035183	.007	.0182
3-7 days	152	119649	0.0012704	.0051	.0263	120	112849	0.0010634	.0043	.0224
7-14 days	96	207511	0.0004626	.0032	.0294	95	195939	0.0004848	.0034	.0257
14-21 days	83	205818	0.0004033	.0028	.0322	77	194441	0.0003960	.0028	.0284
21-28 days	26	202798	0.0001282	.0009	.033	50	191606	0.0002610	.0018	.0302
**Total**	**1011**	**826603**				**868**	**780340**			

STROBE Statement—Checklist of items that should be included in reports of ***cohort studies***

	Item No	Recommendation
**Title and abstract**	1	(*a*) Indicate the study's design with a commonly used term in the title or the abstract
		(*b*) Provide in the abstract an informative and balanced summary of what was done and what was found
**Introduction**		
Background/rationale	2	Explain the scientific background and rationale for the investigation being reported
Objectives	3	State specific objectives, including any prespecified hypotheses
**Methods**		
Study design	4	Present key elements of study design early in the paper
Setting	5	Describe the setting, locations, and relevant dates, including periods of recruitment, exposure, follow-up, and data collection
Participants	6	(*a*) Give the eligibility criteria, and the sources and methods of selection of participants. Describe methods of follow-up
		(*b*) For matched studies, give matching criteria and number of exposed and unexposed
Variables	7	Clearly define all outcomes, exposures, predictors, potential confounders, and effect modifiers. Give diagnostic criteria, if applicable
Data sources/ measurement	8*	For each variable of interest, give sources of data and details of methods of assessment (measurement). Describe comparability of assessment methods if there is more than one group
Bias	9	Describe any efforts to address potential sources of bias
Study size	10	Explain how the study size was arrived at
Quantitative variables	11	Explain how quantitative variables were handled in the analyses. If applicable, describe which groupings were chosen and why
Statistical methods	12	(*a*) Describe all statistical methods, including those used to control for confounding
		(*b*) Describe any methods used to examine subgroups and interactions
		(*c*) Explain how missing data were addressed
		(*d*) If applicable, explain how loss to follow-up was addressed
		(*e*) Describe any sensitivity analyses
**Results**		
Participants	13*	(a) Report numbers of individuals at each stage of study—eg numbers potentially eligible, examined for eligibility, confirmed eligible, included in the study, completing follow-up, and analysed
		(b) Give reasons for non-participation at each stage
		(c) Consider use of a flow diagram
Descriptive data	14*	(a) Give characteristics of study participants (eg demographic, clinical, social) and information on exposures and potential confounders
		(b) Indicate number of participants with missing data for each variable of interest
		(c) Summarise follow-up time (eg, average and total amount)
Outcome data	15*	Report numbers of outcome events or summary measures over time
Main results	16	(*a*) Give unadjusted estimates and, if applicable, confounder-adjusted estimates and their precision (eg, 95% confidence interval). Make clear which confounders were adjusted for and why they were included
		(*b*) Report category boundaries when continuous variables were categorized
		(*c*) If relevant, consider translating estimates of relative risk into absolute risk for a meaningful time period

For peer review only

Other analyses	17	Report other analyses done—eg analyses of subgroups and interactions, and sensitivity analyses
**Discussion**		
Key results	18	Summarise key results with reference to study objectives
Limitations	19	Discuss limitations of the study, taking into account sources of potential bias or imprecision. Discuss both direction and magnitude of any potential bias
Interpretation	20	Give a cautious overall interpretation of results considering objectives, limitations, multiplicity of analyses, results from similar studies, and other relevant evidence
Generalisability	21	Discuss the generalisability (external validity) of the study results
**Other information**		
Funding	22	Give the source of funding and the role of the funders for the present study and, if applicable, for the original study on which the present article is based

*Give information separately for exposed and unexposed groups.

Note: An Explanation and Elaboration article discusses each checklist item and gives methodological background and published examples of transparent reporting. The STROBE checklist is best used in conjunction with this article (freely available on the Web sites of PLoS Medicine at http://www.plosmedicine.org/, Annals of Internal Medicine at http://www.annals.org/, and Epidemiology at http://www.epidem.com/). Information on the STROBE Initiative is available at http://www.strobe-statement.org.

For peer review only

BMJ Open

Does higher early neonatal mortality in boys reverse over the neonatal period? A pooled analysis from three trials of Nepal

Journal:	*BMJ Open*
Manuscript ID	bmjopen-2021-056112.R1
Article Type:	Original research
Date Submitted by the Author:	11-Mar-2022
Complete List of Authors:	Subedi, Seema; Johns Hopkins University Bloomberg School of Public Health, International Health<br>Katz, Joanne; Johns Hopkins University Bloomberg School of Public Health, International Health<br>Erchick, Daniel; Johns Hopkins University Bloomberg School of Public Health, International Health<br>Verhulst, Andrea; University of Pennsylvania, Population Studies Center<br>Khatry, Subarna; Nepal Nutr Intervent Project Sarlahi<br>Mullany, Luke C.; Johns Hopkins University Bloomberg School of Public Health, International Health<br>Tielsch, James; George Washington University School of Public Health and Health Services, Global Health<br>LeClerq, Steven; Johns Hopkins University Bloomberg School of Public Health, International Health; Nepal Nutr Intervent Project Sarlahi<br>Christian, P; Johns Hopkins University Bloomberg School of Public Health, International Health<br>West, KP; Johns Hopkins University Bloomberg School of Public Health, International Health<br>Guillot, Michel; University of Pennsylvania, Population Studies Center
**Primary Subject Heading**:	Paediatrics
Secondary Subject Heading:	Epidemiology, Public health, Global health, Health policy
Keywords:	EPIDEMIOLOGY, Community child health < PAEDIATRICS, NEONATOLOGY

SCHOLARONE™
Manuscripts

I, the Submitting Author has the right to grant and does grant on behalf of all authors of the Work (as defined in the below author licence), an exclusive licence and/or a non-exclusive licence for contributions from authors who are: i) UK Crown employees; ii) where BMJ has agreed a CC-BY licence shall apply, and/or iii) in accordance with the terms applicable for US Federal Government officers or employees acting as part of their official duties; on a worldwide, perpetual, irrevocable, royalty-free basis to BMJ Publishing Group Ltd ("BMJ") its licensees and where the relevant Journal is co-owned by BMJ to the co-owners of the Journal, to publish the Work in this journal and any other BMJ products and to exploit all rights, as set out in our licence.

The Submitting Author accepts and understands that any supply made under these terms is made by BMJ to the Submitting Author unless you are acting as an employee on behalf of your employer or a postgraduate student of an affiliated institution which is paying any applicable article publishing charge ("APC") for Open Access articles. Where the Submitting Author wishes to make the Work available on an Open Access basis (and intends to pay the relevant APC), the terms of reuse of such Open Access shall be governed by a Creative Commons licence – details of these licences and which Creative Commons licence will apply to this Work are set out in our licence referred to above.

Other than as permitted in any relevant BMJ Author's Self Archiving Policies, I confirm this Work has not been accepted for publication elsewhere, is not being considered for publication elsewhere and does not duplicate material already published. I confirm all authors consent to publication of this Work and authorise the granting of this licence.

For peer review only

Does higher early neonatal mortality in boys reverse over the neonatal period? A pooled analysis from three trials of Nepal

Authors: Seema Subedi[1], Joanne Katz[1], Daniel J. Erchick[1], Andrea Verhulst[2], Subarna K. Khatry[3], Luke C. Mullany[1], James M. Tielsch[4], Steven C. LeClerq[1,3], Parul Christian[1], Keith P. West Jr.[1] , Michel Guillot[2]

Affiliations:

[1] Department of International Health, Johns Hopkins Bloomberg School of Public Health

[2] Department of Sociology & Population Studies Center, University of Pennsylvania

[3] Nepal Nutrition Intervention Project – Sarlahi, Kathmandu, Nepal.

[4] Department of Global Health, George Washington University Milken Institute School of Public Health.

Emails: ssubedi2@jhu.edu, jkatz1@jhu.edu, derchick@jhu.edu, verhulst@sas.upenn.edu, skhatry@wlink.com.np, lmullany@gmail.com, jtielsch@email.gwu.edu, sleclerq@mos.com.np, pchrist1@jhu.edu, west1@jhu.edu, miguillo@sas.upenn.edu

Correspondence: Seema Subedi, MPH. Department of International Health, Johns Hopkins Bloomberg School of Public Health, 615 N. Wolfe Street, Baltimore, MD, 21205, United States. ssubedi2@jhu.edu

Word Count:

Abstract: 298

Main Manuscript (including tables): 4461

For peer review only

ABSTRACT

Objectives

Neonatal mortality is generally 20% higher in males than females due to biological phenomena. Only a few studies have examined more finely categorized age patterns of neonatal mortality by sex, especially in the first few days of life. The objective of this study is to examine sex differentials in neonatal mortality by detailed ages in a low-income setting.

Design

This is a secondary observational analysis of data.

Setting

Rural Sarlahi district, Nepal.

Participants

Neonates born between 1999 and 2017 in three randomized controlled trials.

Outcome Measures

We calculated study specific and pooled mortality rates for males and females by ages (0-1, 1-3, 3-7, 7-14, 14-21 and 21-28 days) and estimated hazard ratios (HR) using Cox proportional hazard models for male versus female mortality for treatment and control groups together (n=59,729).

Results

Neonatal mortality was higher in males than females in individual studies: 44.2 vs. 39.7 in males and females in 1999-2000; 30.0 vs. 29.6 in 2002-2006; 33.4 vs. 29.4 in 2010-2017; and 33.0 vs. 30.2 in the pooled data analysis. Pooled data found that early neonatal mortality (HR=1.17; 95% CI: 1.06-1.30) was significantly higher in males than females. All individual datasets showed a reversal in mortality by sex after the 3rd week of life. In the 4th week, a reversal was observed, with mortality in females 2.43 times higher than males (HR=0.41; 95% CI: 0.31-0.79).

Conclusions:

Males had higher mortality in the first week followed by no sex difference in weeks 2 and 3 and a reversal in risk in week 4, with females dying at more than twice the rate of males. This may be a result of gender discrimination and social norms in this setting. Interventions to reduce gender discrimination at the household level may reduce female neonatal mortality.

Trial Registration Number: NCT00115271, NCT00109616, NCT01177111

For peer review only - http://bmjopen.bmj.com/site/about/guidelines.xhtml

For peer review only

STRENGTHS AND LIMITATIONS OF THE STUDY

- Since the neonates were followed at frequent intervals, we could examine the sex differentials in neonatal mortality at more detailed age (0-1, 1-3, 3-7, 7-14, 14-21, and 21-28 days), which have not been seen in other studies.
- Since we used data from three different trials in the same settings, it was appropriate to analyze by pooling the data.
- We could not examine the determining factors for the main result of the study and our discussions are based on the existing literature.

For peer review only

INTRODUCTION

Since the 1960s, high-income countries (HICs) have reported higher neonatal mortality rates in males than females.[1-6] For overall neonatal mortality, males are at an approximately 20% greater risk of neonatal mortality than females. These differences are explained primarily as biologically driven phenomenon with the predominant causes of death being non-infectious.[2, 3, 5, 6] Several factors associated with higher neonatal mortality in males include intrauterine growth restriction, respiratory distress syndrome, prematurity and birth asphyxia.[7-10] Studies examining immunologic differences in animal models have showed that females have stronger innate and humoral responses to infection, making them better able to fight infection.[11-13] These studies also show that there is an association between sex hormones and immune function, where testosterone in males suppresses the immune system, while estradiol and progesterone in females improve both the innate and humoral immune responses.[11-13] Males also have higher birthweights than females, leading to a higher risk of complications during delivery and injuries at birth, although in general, low birthweight is associated with higher mortality.[2, 11, 14-19] Data from HICs show that the mortality in males is higher than females not only during the neonatal period, but also after the neonatal period, through infancy and beyond.[2, 20]

In low- and middle-income countries (LMICs) with higher neonatal mortality (more than 30 per 1,000 live births), sex differences in neonatal mortality have been inconsistent. A multi-country study in Sub-Saharan Africa reported higher neonatal mortality ratios for males to females ranging from 1.1. to 1.6.[21] Similarly, an Indonesian study of Demographic and Health Surveys (DHS) data reported an adjusted sex difference in neonatal mortality of 1.49 times higher in males.[22] However, a Pakistani study reported an overall sex difference of 0.82, indicating higher neonatal mortality risk in females.[23]

Separating neonatal mortality into early and late neonatal mortality, the literature from both HICs and LMICs generally show that males have higher rates of mortality than females in the early neonatal period (first week of life).[4, 5, 19, 23, 24] The extent of these differences varies by factors like level of neonatal mortality, causes of neonatal mortality, and other region-specific factors. However, sex differences in mortality during the late neonatal period have not been consistent.

Evidence from South Asia has suggested that although males are at higher risk of death in the early neonatal period, this pattern can reverse in the late neonatal period.[23, 24] A study by Rosenstock et al., which used one of the datasets of our analysis (Chlorhexidine Study), showed there was a reversal in the mortality pattern by sex in Nepal.[19] In the early neonatal period, males were at 20% higher mortality risk, assumed to be due to biological factors, whereas in the late neonatal period, girls were at a 43% higher mortality risk. This was associated with ethnicity and the gender structure of siblings in the family rather than by gender preference alone, where girls born to families with only girls had higher risk.[19] In an urban Pakistani study, where overall neonatal mortality was lower in males (0.82), the sex differences in early and late neonatal mortality were 1.21 and 0.28 respectively, indicating a reversal of risk in the later weeks of the neonatal period.[23] Differential health care-seeking behaviors and gender preference for male infants, have been reported as explanations for higher late neonatal and infant mortality in females.[23-28]

Some South Asian studies have examined sex differences in post-neonatal mortality. An analysis of data from a randomized trial in rural northern India comparing sex differences in mortality during the neonatal period and beyond showed that males had 1.25 times higher neonatal mortality in the 1st week of life. In the post-neonatal period, however, females had significantly higher mortality; 1.4 and 1.7 times higher in days 29-180 and days 181-365, respectively.[15] Factors associated with excess female mortality in the post-neonatal period were caste and mother’s occupation (higher for mothers working outside the home).

A recent study with 297,509 live births in India and Pakistan showed that both overall and early neonatal mortality risk were significantly higher in males than females. However, there was no significant difference by sex in late neonatal mortality, and mortality between 29-42 days.[7]

For peer review only - http://bmjopen.bmj.com/site/about/guidelines.xhtml

Given that some South Asian countries showed a reversal in neonatal mortality, and others have not, we examined data from three, sequential, community-based randomized controlled trials (RCTs) conducted in the District of Sarlahi located in the east-central, southern rural plains (Terai) of Nepal. The district is in the rural low-lying area of Nepal that borders the Indian state of Bihar. This area has two main ethnic groups, Pahadi or people of hill origin, and Madeshi, who are from the plains. Health indicators and access to care has changed from 1999 through 2017. In the first trial, only 5% of women delivered in a facility, 9% in the second trial (2002-2006), and 42% in the third trial (2010-2017). [29,30, personal communication (Katz)] This increase in facility delivery coincided with a government cash incentive scheme that paid women to get 4 antenatal care visits and delivery in a facility. Maternal literacy increased from 20% to 25% to 32% in these trials, and mean birthweight from 2616g to 2705g to 2773g. [29,30, personal communication (Katz)] These studies included frequent in-person follow-up of all live born infants with exact date of deaths, allowing us to analyze sex differences in mortality by more finely categorized ages (0-1 day, 1-3 days, 3-7 days, 7-14 days, 14-21 days, and 21-28 days). The objective of this study is to examine sex differentials in neonatal mortality by detailed ages using data in a low-income setting. This can help us pinpoint the age at which the pattern of sex difference in mortality changes or reverses, which could help us plan interventions accordingly.

For peer review only - http://bmjopen.bmj.com/site/about/guidelines.xhtml

METHODS

This is a secondary observational analysis of data from three randomized controlled trials. Characteristics of the datasets used in this analysis are provided in Table 1. The studies provide pregnancy cohorts from 1999 through 2017. Child follow-up duration ranged from 28 days to 5 years. All studies were community based RCTs conducted in the same rural community of Nepal by the Nepal Nutrition Intervention Project, Sarlahi (NNIPS). The first study, NNIPS-3, followed pregnancies and births in the study area from 1999-2000, to look at the effect of antenatal multiple micronutrient supplementation on birth outcomes and the health of their children (Clinicaltrials.gov (NCT00115271)). [29] The second, the Chlorhexidine Study (CHX), followed participants from 2002-2006 to assess if a chlorhexidine body wipe and/or chlorhexidine application to the umbilical stump reduced neonatal mortality (Clinicaltrials.gov (NCT00109616)).[30] The third, the Nepal Oil Massage Study (NOMS), followed participants from 2010-2017 to evaluate the impact of sunflower versus mustard seed oil massage on neonatal mortality (Clinicaltrials.gov (NCT01177111)). In each study, vital status of newborns at birth and through 28 days of life was ascertained in a prospective follow-up done by study teams and date of death was recorded. In the NNIPS-3 and CHX studies, live vs stillbirths were self-reported by mothers. In NOMS, infants were considered born alive if the baby moved, cried or breathed after the birth.

Individual-level data for each pregnancy and live birth included date and type (live/stillbirth) of outcome, date of death, length of follow-up, sex of the infant, and whether the birth was a singleton or multiple. Only live births were used for this analysis. For each individual dataset, we calculated survival times for live births using dates of birth and death. Survival times were split into age categories (0-1 (first 24 hours), 1-3, 3-7, 7-14, 14-21, and 21-28 days). The total deaths and person-time in each category were used to calculate death rates, and probability of dying with 95% CI in those groups, separately for males and females. Differences in the probability of dying between males and females were visualized using mortality curves. Cox regression was used to estimate hazard ratios with 95% CI for male versus female mortality for overall neonatal mortality (0-28 days), early neonatal mortality (0-7 days), late neonatal mortality (7-28 days), and for the more finely categorized age groups described above. No covariates other than sex were included in the Cox regression. No adjustments were made for time-trends in neonatal mortality rates. The aim of this analysis was not to explain drivers of neonatal mortality trends but rather to compare the differential neonatal survival by sex within the same time periods. Datasets were then pooled

For peer review only

to conduct the same analyses. Data were analyzed by combining intervention and control groups after fitting a Cox regression model for each study and a pooled model, with an interaction between sex and a binary intervention indicator , which found no significant interaction effects (Hazard Ratio (HR) 0.76, 95% Confidence Interval (CI) 0.37, 1.55; HR 1.08, (0.80, 1.47); HR 1.14 (0.89, 1.47); HR 1.07 (0.89, 1.29) for NNIPS-3, CHX, NOMS and pooled analysis respectively.

The research proposal (IRB Protocol number 827014, and IRB number 8) was considered exempt by the Institutional Review Board at the University of Pennsylvania, authorized by 45 CFR 46.101, category 4. NNIPS-3, CHX and NOMS studies were approved by the institutional review board (IRB) of the Johns Hopkins Bloomberg School of Public Health. NNIPS-3 and CHX were approved by the IRB of the Institute of Medicine, Tribhuvan University in Nepal. NOMS was approved by the Nepal Health Research Council in Nepal. Verbal consent was obtained from women for their participation and their infants, for all studies.

Patient and Public Involvement Statement

Patients or the public were not involved in the design, conduct, reporting, or dissemination plans for this research.

For peer review only

For peer review only - http://bmjopen.bmj.com/site/about/guidelines.xhtml

Table 1. Methodologies of studies included in the analysis of sex specific mortality by age.

Study	Study Design	Birth Cohorts	Total FUP time	Neonatal FUP visits	Neonatal-level Intervention	Total LB in analysis	Number of Neonatal Deaths	Cumulative Neonatal Mortality (per 1000 LBs)
**NNIPS-3**	Randomized Controlled Community Trial	1999-2000	28 days *	Day 1, 2, 3, 4, 5, 6, 7, 8, 9, 10, 17, 24 and 31.	None	M=2,082	M= 92	M=44.2
						F= 2,045	F=81	F=39.7
						**Total= 4,127**	**Total=173**	**Overall=41.9**
**CHX Study**	Randomized Controlled Community Trial	2002-2006	28 days	Day 1, 2, 3, 4, 6, 8, 10, 12, 14, 21 and 28.	Chlorhexidine and Placebo wipe	M= 12,188	M=371	M=30.5
						F= 11,456	F=338	F=29.6
						**Total=23,644**	**Total=709**	**Overall=30.0**
**NOMS**	Randomized Controlled Community Trial	2010-2017	28 days	Day 1, 3, 7, 10, 14, 21 and 28.	Sunflower Oil and Mustard Oil massage	M=16,533	M=548	M=33.4
						F=15,425	F=449	F=29.4
						**T= 31,958**	**T=997**	**Overall=31.4**
**Total**						**59,729**	**1,879**	**31.6**

**In NNIPS-3 follow-up went beyond 28 days, but for the other 2 studies, it went through 28 days only.*

For peer review only

RESULTS

The overall neonatal mortality risk was higher in males than females in the individual studies as well as the pooled analysis (N=59,729 live births) (Table 1). Neonatal mortality was 41.9 per 1,000 live births (LB) (44.2 versus 39.7 in males and females), 30.0 per 1,000 LB (30.5 versus 29.6 in males and females), 31.4 per 1,000 LB (33.4 versus 29.4 in males and females) and 31.6 per 1000 LB (33.0 versus 30.2 in males and females) in 1999, 2002, 2010 and the pooled analysis respectively. Child's sex was missing for a very small number of neonatal deaths (1/174 in NNIPS-3, none in CHX study, and 4/1001 in NOMS).

The 1999 NNIPS-3 study found that more males than females died early (0-1 day, 1-3 days), then the rates for males and females converged, until a reversal was seen in the 4th week of life (Figure 1), (HR= 0.39; 95% CI: 0.08-2.03). The 2002 Chlorhexidine study found males had a higher mortality than females in the early neonatal period (0-1 day, 1-3 days and 3-7 days), then mortality quickly reversed after the first week and continued until the 4th week (Figure 1), (HR= 0.49; 95%CI: 0.24-0.98). The 2010 NOMS study had higher mortality in males than females in the early neonatal period (0-1 day, 1-3 days and 3-7 days), then mortality converged in the 2nd and 3rd weeks, followed by a reversal after the 3rd week (Figure 1), (HR= 0.51; 95% CI: 0.25-1.04). A common finding in all three studies was that there was a reversal after the 3rd week of life, where female mortality was higher than for males, although this reversal was statistically significant only in the CHX study (Table 2). Our pooled analysis showed mortality among males was higher through the 2nd week (0-1 day, 1-3 days, 3-7 days and 7-14 days), followed by similar rates during the 3rd week (14-21 days), followed by a statistically significant reversal in the 4th week of life (21-28 days) (Figure 2). For the pooled analysis, results from Cox regression showed that early neonatal mortality (HR=1.17; 95% CI: 1.06-1.30) was significantly higher in males than females, and the 4th week mortality reversed with 2.43 (95% CI: 1.26-3.33) times higher in females than males (HR= 0.41; 95% CI: 0.31-0.79) (Table 3). The details of the mortality rates by age and sex, including age group, deaths, person year, death rate, and probability of dying for NNIPS-3 study, Chlorhexidine study, NOMS study and pooled analysis are shown in the Annex Table 1, 2, 3 and 4 respectively.

For peer review only

Table 2: Hazard Ratio (Male/Female) for Neonatal Mortality for Individual Studies

	NNIPS-3 (1999-2000) N=4127			CHX Study (2002-2006) N=23,644			NOMS (2010-2017) N=31,958		
**Age Category**	**Hazard Ratio (M/F)**	**95%CI**	**p-value**	**Hazard Ratio (M/F)**	**95%CI**	**p-value**	**Hazard Ratio (M/F)**	**95%CI**	**p-value**
Overall Neonatal (0-28days)	**1.11**	0.83, 1.51	0.458	**1.03**	0.89, 1.20	0.652	**1.14**	1.01, 1.29	0.037
Early Neonatal (0-7 days)	**1.22**	0.85, 1.75	0.272	**1.17**	0.99, 1.40	0.067	**1.16**	1.02, 1.34	0.029
Late Neonatal (7-28 days)	**0.91**	0.53, 1.57	0.744	**0.71**	0.53, 0.96	0.024	**1.04**	0.79, 1.38	0.777
**Sub-analysis by days from birth**									
0-1 day	**1.37**	0.82, 2.30	0.224	**1.18**	0.92, 1.52	0.18	**1.11**	0.92, 1.35	0.284
1-3 days	**1.82**	0.93, 3.58	0.081	**1.13**	0.84, 1.52	0.415	**1.16**	0.90, 1.50	0.247
3-7 days	**0.49**	0.21, 1.16	0.105	**1.22**	0.82, 1.81	0.316	**1.35**	0.98, 1.89	0.068
7-14 days	**0.91**	0.43, 0.95	0.819	**0.85**	0.55, 1.32	0.482	**1.08**	0.70, 1.68	0.705
14-21 days	**1.23**	0.49, 3.12	0.658	**0.68**	0.41, 1.13	0.135	**1.33**	0.86, 2.10	0.198
21-28 days	**0.39**	0.08, 2.03	0.267	**0.49**	0.24, 0.98	0.046	**0.51**	0.25, 1.04	0.063

NNIPS – Nepal Nutrition Intervention Project Sarlahi, CHX – Chlorhexidine intervention trial, NOMS – Nepal Oil Massage Study

For peer review only

For peer review only - http://bmjopen.bmj.com/site/about/guidelines.xhtml

Table 3: Probability of Dying and Hazard Ratio (Male/Female) of Neonatal Mortality for Pooled Analysis

POOLED (1999-2017) N=59,729

	**Males(N=30,803)**			**Females (N=28,926)**			**Hazard**		
**Age Category**	**Deaths**	**Person Year**	**Probability of Dying**	**Deaths**	**Person Year**	**Probability of Dying**	**Ratio (M/F)**	**95%CI**	**p-value**
Overall Neonatal (0-28days)	1011	826,603	.0086	868	780,341	.0078	1.09	1.00, 1.20	0.045
Early Neonatal (0-7 days)	806	210,476	.0268	646	198,355	.0228	1.17	1.06, 1.30	0.002
Late Neonatal (7-28 days)	205	616,127	.0023	222	581,986	.0027	0.87	0.72, 1.05	0.158
**Sub-analysis by days from birth**									
0-1 day	401	30,472	.0132	326	28,660	.0114	1.15	1.00, 1.34	0.051
1-3 days	253	60,356	.0084	200	56,846	.0070	1.19	0.99, 1.43	0.064
3-7 days	152	119,649	.0051	120	112,849	.0043	1.19	0.94, 1.52	0.146
7-14 days	96	207,511	.0032	95	195,939	.0034	0.94	0.72, 1.27	0.745
14-21 days	83	205,818	.0028	77	194,441	.0028	1.01	0.75, 1.39	0.909
21-28 days	26	202,798	.0009	50	191,606	.0018	0.41	0.31, 0.79	0.003

For peer review only

DISCUSSION

Our study found higher mortality in males than females early in the neonatal period followed by a reversal in the 4th week of life in each of the individual studies. This work extends that of Rosenstock et al. to include a longer time span from 1999 through 2017 in the same geographic area.[19] In the pooled analysis, this reversal was statistically significant and the mortality hazard ratio was 2.43 times higher in females than males in the 4th week of life. This is similar to the findings by Rosenstock et al. in rural Nepal and Jehan et al. in urban Pakistan although they compared only early and late neonatal mortality and found a reversal in the late neonatal period. [19, 23] However, a recent study in India and Pakistan showed higher male mortality in the early neonatal period, but no significant difference in male and female mortality in the late neonatal period or between 29-42 days.[7] In that study, the first follow-up was within 48 hours of delivery, with one more visit at 42-day post-partum.[7] Our analysis had more frequent visits and a prospective record of exact date of death.

Another large randomized controlled trial rural North India, which examined differences in the post-neonatal period, found a reversal in 29-180 and 181-365 days after birth.[15] They followed live births on day 29 after the infant's birth and at ages 3, 6, 9 and 12 months to obtain vital status of the infant.[15] For this reason, they analyzed the difference in the post-neonatal period but could not do so in the late neonatal period. Our study with rigorous follow-up within the neonatal period allowed us to examine the sex differences in more finely categorized ages and identify that the reversal took place as early as the 4th week of life.

Our previous work with the 2002-2006 dataset further examined possible reasons for sex differences in mortality, finding that ethnicity, differential neonatal care seeking behavior and prior family composition with multiple daughters were important factors associated with higher late neonatal mortality in females.[19, 31] While socioeconomic conditions improved over the time period from 1999 to 2017, the only major changes in health care during the time period of the three studies was an increase in facility delivery, particularly in the trial that spanned 2010-2017, due to the government cash incentive program. However, there was no evidence that the differential survival of neonates by sex varied over this time period. In Nepal, gender discrimination originates at the household level. Since the 1980s, when the World Fertility Surveys first documented evidence of son preference, Nepal has been categorized as a country with a high level of this preference. [32] This practice is still common,

For peer review only - http://bmjopen.bmj.com/site/about/guidelines.xhtml

as seen in a 2012 survey on 1,000 Nepalese men aged 18-49 showing that the majority (90%) believed that a man with only daughters is unfortunate and not having a son reflects a lack of moral virtue. Nearly half said that a woman's important roles are limited to taking care of her home and cooking for her family. Married women reported that maintaining an income-generating job is precluded by care-giving for small children (32%), lack of permission from other household/family decision makers (19%), and the workload at home (18%). Only 26% of married Nepalese women reported making independent decisions regarding their own health care.[33] Many women are still restricted to the private sphere with unpaid work. Women work for an estimated average of 268 minutes a day on household chores, whereas men work only 56 minutes.[34] This deprives women of quality education, awareness and exposure. Socially, sons are given preference because of the various cultural and economic roles that are believed to be performed by sons only: performing lighting of the funeral pyre, continuing the family lineage and providing old age economic security for their parents, whereas girls are considered an economic liability because they have to live in their parents' home until marriage, for which a dowry must be provided to the groom's family. Upon marriage they become part of the economy of the husband's family, hence being an economic drain on the family from birth onwards.[35] Nepal's patrilineal and patrilocal social structure combined with socio-economic and religious values leads to son preference and gender discrimination.

A systematic review found higher care seeking behavior for male than female neonates in seventeen studies in South Asia, particularly in households with older female siblings.[36] In addition, for male babies, care-seeking was more frequent, from better qualified care providers, and with higher expenditure compared to females. Studies also have consistently shown that households with female children were more likely to report discrimination, because family members perceived that care for illness was not so important, leading to reduced care-seeking.[36] Similar to Nepal, a 2011 UNICEF report on China also indicated that the discrimination against female infants was highest for those who had older female siblings.[37]

In Nepal, where most children are exclusively breastfed, the median duration of breastfeeding for males and females are 4.2 months and 4.1 months. [38] Hence this is an unlikely explanation for the reversal of mortality in the 4th week of life. The differential may be explained by

For peer review only - http://bmjopen.bmj.com/site/about/guidelines.xhtml

For peer review only

poorer nutrition, care and rest provided to mothers giving birth to daughters, son preference being the root cause of discrimination in the family. It could also point to specific parental behaviors that takes place starting at that age and suggests a critical age window for intervention. However, the 3-week threshold could also just be an indication that the gender discrimination starts from birth or even earlier, but takes at least three weeks for the biological and natural survival advantage of females to be overcome by the social advantage of males. Further studies could explore more about why this reversal starts specifically in the 4th week of life.

Neonatal mortality in Nepal has continued to decrease from 39 to 21 per 1,000 live births, with male neonatal mortality decreasing from 52 to 24 per 1000 LB (reduction of 28%) and female neonatal mortality decreasing from 43 to 17 per 1000 LB (reduction of 26%) from 2001 to 2016.[33, 38] Given that males have a biological disadvantage in neonatal survival, one could have expected female neonatal mortality to have decreased more than for males. If female mortality could decrease more than it has, this could contribute to a greater reduction in overall neonatal mortality. Although neonatal mortality in Nepal was decreasing from 2001 to 2016, it still contributed to a higher percentage of under-5 child mortality because mortality among older children has decreased faster than neonatal mortality.[39] If this trend continues, the Sustainable Development Goals (SDG) target of reducing neonatal mortality to 12 per 1,000 live births by 2030 in Nepal will be difficult to achieve.[40] Therefore, a focus on reducing female neonatal mortality could help meet the SDG for neonatal mortality and for gender equity. A cross national study from 138 countries also showed evidence that the Gender Inequality Index (GII) was positively associated with neonatal mortality.[41] Applying interventions to address gender discrimination by addressing cultural and social barriers at the household level may help reduce neonatal mortality.

In the early neonatal period, preterm birth is one of the main causes of deaths, while in the late neonatal period, sepsis and pneumonia account for more deaths.[42, 43] Studies also show that preterm birth is higher in males than females.[44-46] Given the biological susceptibility of males towards more early neonatal deaths both in the LMICs and HICs, it is not as easy to intervene. However, the main causes of late neonatal mortality like sepsis and pneumonia can be intervened on through improved care-seeking practices. So, if male and female children are provided with similar care-seeking practices in the late neonatal period, the sex-differences in neonatal mortality might be reduced, as in HICs.

For peer review only

Gender discrimination not only affects the quality of life of girls and women, but also reduces their survival in the neonatal period. Interventions to strengthen gender equality, such as counselling to woman and their family during antenatal care and postnatal care visits may be helpful to improve female and male neonatal survival. Since the reversal takes place during 4th week of life, specific counselling interventions to parents and family could be targeted in the first 3 weeks of child's life.

The strength of this study is that it incorporates data from sequential randomized community trials from the same site sharing many similar field procedures carried out by the same, highly trained field teams over a 15-year period, so that pooling is reasonable and allows for a more precise analysis of sex differences in neonatal mortality. In addition, these studies have enrollment from pregnancy, which reduces the likelihood that early child deaths have been missed. Neonatal deaths have been tracked at frequent intervals to improve accuracy of age at death, enabling us to conduct survival analysis and Cox regression to obtain improved mortality estimates and hazard ratios.

This study was not able to examine the specific reasons for the mortality reversal. However, we have discussed possible reasons based on the existing literature. Further studies could examine why this reversal takes place as early as the 4th week of life and whether this reversal persists beyond the neonatal period.

CONCLUSION

Male mortality is higher than female in the early neonatal period, a biological phenomenon seen worldwide. However, this natural pattern is quickly reversed after the 3rd week of life in Nepal. This is likely due to gender discrimination and social norms that operate at household level. Implementing interventions to reduce gender discrimination at the household level could prevent this reversal and decrease female neonatal mortality, thereby reducing overall neonatal mortality and improving gender equity.

For peer review only - http://bmjopen.bmj.com/site/about/guidelines.xhtml

OTHER INFORMATION

Ethics approval

The research proposal (IRB Protocol number 827014, and IRB number 8) was considered exempt by the Institutional Review Board at the University of Pennsylvania, authorized by 45 CFR 46.101, category 4.

Consent for publication:

Not applicable.

Data Sharing:

No additional data available.

Competing interests:

The authors declare that they have no competing interests.

Funding source:

This work was supported by the National Institute for Child Health and Human Development (NICHD 1R01HD090082-01 and R01HD092411). NNIPS-3 was supported by Cooperative agreement HRN-A-00-97-00015-00 between Office of Health and Nutrition, US Agency for International Development (USAID), Washington, DC with additional support from the UNICEF Country Office, Kathmandu, Nepal (Grant number NA), and grant GH614 from the Bill & Melinda Gates Foundation (Seattle, WA). The CHX study was supported by R01HD044004 and R01HD038753 from the National Institutes of Health (Bethesda, MD); grant 810–2054 from the Bill and Melinda Gates Foundation (Seattle, WA) and Cooperative Agreements HRN-A-00-97-00015-00 and GHS-A-00-03-000019-00 between Johns Hopkins University and the Office of Health and Nutrition, US Agency for International Development (Washington, DC.). NOMS was supported by the National Institutes for Child Health and Development (HD060712) and the Bill & Melinda Gates Foundation (OPP1084399). The funders did not have a role in the design of the study, the data collection, nor the analysis, interpretation and writing of the manuscript.

For peer review only

Authors' contribution:

SS, JK, DE, AV, MG conceptualized and designed the study. SS conducted the analysis and wrote the manuscript. SK, LM, JT, SL, PC and KW were the investigators of the three different trials used in the paper, and along with other authors, they have reviewed results, discussed interpretations, and contributed to development and revision of the manuscript.

Acknowledgements:

We thank the women, infants, and their families who participated in this study, and the NNIPS project staff in Nepal. We are also grateful to Mrs. Lee S-F Wu from Johns Hopkins Bloomberg School of Public health for her contribution to the NNIPS-3 study.

REFERENCES

1 Elsmén E, Steen M, Hellström-Westas L. Sex and gender differences in newborn infants: why are boys at increased risk?. *Journal of Men's Health and Gender* 2004;1:303-11.

2 Drevenstedt GL, Crimmins EM, Vasunilashorn S, et al. The rise and fall of excess male infant mortality. *Proceedings of the National Academy of Sciences* 2008;105:5016-21.

3 Nathanson CA. Sex differences in mortality. *Annual review of sociology* 1984;10:191-213.

4 Hammoud EI. Studies in fetal and infant mortality. II. Differentials in mortality by sex and race. *American Journal of Public Health and the Nations Health* 1965;55:1152-63.

5 Waldron I. Patterns and causes of excess female mortality among children in developing countries. *World health statistics quarterly 1987; 40 (3): 194-210* 1987.

6 Gove WR, Carpenter GR. The fundamental connection between nature and nurture: a review of the evidence: Free Press 1982.

7 Aghai ZH, Goudar SS, Patel A, et al. Gender variations in neonatal and early infant mortality in India and Pakistan: a secondary analysis from the Global Network Maternal Newborn Health Registry. *Reproductive Health* 2020;17:1-11.

8 Hou L, Wang X, Li G, et al. Cross sectional study in China: fetal gender has adverse perinatal outcomes in mainland China. *BMC pregnancy and childbirth* 2014;14:372.

9 Aibar L, Puertas A, Valverde M, et al. Fetal sex and perinatal outcomes. *J Perinat Med* 2012;40:271-6.

10 Simchen MJ, Weisz B, Zilberberg E, et al. Male disadvantage for neonatal complications of term infants, especially in small-for-gestational age neonates. *The Journal of Maternal-Fetal & Neonatal Medicine* 2014;27:839-43.

For peer review only - http://bmjopen.bmj.com/site/about/guidelines.xhtml

11 Klein SL. Hormones and mating system affect sex and species differences in immune function among vertebrates. *Behav Processes* 2000;51:149-66.

12 Klein SL, Marson AL, Scott AL, et al. Neonatal sex steroids affect responses to Seoul virus infection in male but not female Norway rats. *Brain Behav Immun* 2002;16:736-46.

13 Klein SL. Hormonal and immunological mechanisms mediating sex differences in parasite infection. *Parasite Immunol* 2004;26:247-64.

14 Mishra SK, Ram B, Singh A, et al. Birth order, stage of infancy and infant mortality in India. *J Biosoc Sci* 2018;50:604-25.

15 Chowdhury R, Taneja S, Mazumder S, et al. Gender differences in infant survival: a secondary data analysis in rural North India. *BMJ open* 2017;7.

16 Yorita KL, Holman RC, Sejvar JJ, et al. Infectious disease hospitalizations among infants in the United States. *Pediatrics* 2008;121:244-52.

17 Elsmén E, Pupp IH, Hellström-Westas L. Preterm male infants need more initial respiratory and circulatory support than female infants. *Acta Paediatrica* 2004;93:529-33.

18 Baqui AH, Darmstadt GL, Williams EK, et al. Rates, timing and causes of neonatal deaths in rural India: implications for neonatal health programmes. *Bull World Health Organ* 2006;84:706-13.

19 Rosenstock S, Katz J, Mullany LC, et al. Sex differences in neonatal mortality in Sarlahi, Nepal: the role of biology and environment. *J Epidemiol Community Health* 2013;67:986-91.

20 Zhao D, Zou L, Lei X, et al. Gender differences in infant mortality and neonatal morbidity in mixed-gender twins. *Scientific reports* 2017;7:1-6.

21 Garenne M. Sex differences in health indicators among children in African DHS surveys. *J Biosoc Sci* 2003;35:601-14.

22 Titaley CR, Dibley MJ, Agho K, et al. Determinants of neonatal mortality in Indonesia. *BMC Public Health* 2008;8:232.

23 Jehan I, Harris H, Salat S, et al. Neonatal mortality, risk factors and causes: a prospective population-based cohort study in urban Pakistan. *Bull World Health Organ* 2009;87:130-8.

24 Katz J, West Jr KP, Khatry SK, et al. Risk factors for early infant mortality in Sarlahi district, Nepal. *Bull World Health Organ* 2003;81:717-25.

25 Nielsen BB, Liljestrand J, Hedegaard M, et al. Reproductive pattern, perinatal mortality, and sex preference in rural Tamil Nadu, south India: community based, cross sectional study. *BMJ* 1997;314:1521.

For peer review only

26 Willis JR. Perceived neonatal morbidities and healthcare utilization for neonatal health in rural Uttar Pradesh, India: effects of a behavior-change communication intervention: ProQuest 2007.
27 Pandey A, Sengupta PG, Mondal SK, et al. Gender differences in healthcare-seeking during common illnesses in a rural community of West Bengal, India. *Journal of Health, Population and Nutrition* 2002:306-11.
28 Howlader AA, Bhuiyan MU. Mothers' health-seeking behaviour and infant and child mortality in Bangladesh. *Asia-Pacific Population Journal* 1999;14:59.
29 Christian P, West KP, Khatry SK, et al. Effects of maternal micronutrient supplementation on fetal loss and infant mortality: a cluster-randomized trial in Nepal. *Am J Clin Nutr* 2003;78:1194-202.
30 Tielsch JM, Darmstadt GL, Mullany LC, et al. Impact of newborn skin-cleansing with chlorhexidine on neonatal mortality in southern Nepal: a community-based, cluster-randomized trial. *Pediatrics* 2007;119:e330-40.
31 Rosenstock S, Katz J, Mullany LC, et al. Sex differences in morbidity and care-seeking during the neonatal period in rural southern Nepal. *Journal of Health, Population and Nutrition* 2015;33:1-10.
32 Cleland, J, Verall, J., and Vasessen, M. Sex of Children and their Influence on Reproductive Behavior. World Fertility Survey Comparative Studies 1983.
33 Ministry of Health - MOH/Nepal, New ERA/Nepal, ICF. Nepal Demographic and Health Survey 2016 2017.
34 Budlender D. Moussié R. Making Care Visible Women's Unpaid Care Work in Nepal, Nigeria, Uganda and Kenya 2013.
35 Nanda P, Gautam A, Verma R, et al. Study on gender, masculinity and son preference in Nepal and Vietnam: International Center for Research on Women (ICRW) New Dehli 2012.
36 Ismail SA, McCullough A, Guo S, et al. Gender-related differences in care-seeking behaviour for newborns: a systematic review of the evidence in South Asia. *BMJ global health* 2019;4:e001309.
37 UNICEF. Boys and girls in the life cycle. 2011.
38 Family Health Division of the Department of Health Services of the Ministry of Health/Nepal, New ERA/Nepal, Macro O. Nepal Demographic and Health Survey 2001 2002.

For peer review only - http://bmjopen.bmj.com/site/about/guidelines.xhtml

39 Paudel, D., A. Thapa, P. R. Shedain, and B. Paudel. Trends and determinants of neonatal mortality in Nepal: Further analysis of the Nepal Demographic and Health Surveys, 2001-2011. 2013.

40 National Planning Commission. Sustainable Development Goals 2016–2030: National (Preliminary) Report. *Kathmandu: National Planning Commission* 2015.

41 Brinda EM, Rajkumar AP, Enemark U. Association between gender inequality index and child mortality rates: a cross-national study of 138 countries. *BMC Public Health* 2015;15:97-3 doi:10.1186/s12889-015-1449-3 [doi].

42 Dandona R, Kumar GA, Bhattacharya D, et al. Distinct mortality patterns at 0–2 days versus the remaining neonatal period: results from population-based assessment in the Indian state of Bihar. *BMC medicine* 2019;17:1-19.

43 Sankar MJ, Natarajan CK, Das RR, et al. When do newborns die? A systematic review of timing of overall and cause-specific neonatal deaths in developing countries. *Journal of perinatology* 2016;36:S1-S11.

44 Peelen MJ, Kazemier BM, Ravelli AC, et al. Impact of fetal gender on the risk of preterm birth, a national cohort study. *Acta Obstet Gynecol Scand* 2016;95:1034-41.

45 Zeitlin J, Ancel P, Larroque B, et al. Fetal sex and indicated very preterm birth: Results of the EPIPAGE study. *Obstet Gynecol* 2004;190:1322-5

46 Lao TT, Sahota DS, Suen SS, et al. The impact of fetal gender on preterm birth in a southern Chinese population. *The Journal of Maternal-Fetal & Neonatal Medicine* 2011;24:1440-3.

For peer review only - http://bmjopen.bmj.com/site/about/guidelines.xhtml

Figure 1: Sex difference in probability of dying for individual studies- NNIPS-3, CHX and NOMS (from left to right)

Figure 2: Sex difference in probability of dying for Pooled Analysis, Nepal 1999-2017

For peer review only

For peer review only - http://bmjopen.bmj.com/site/about/guidelines.xhtml

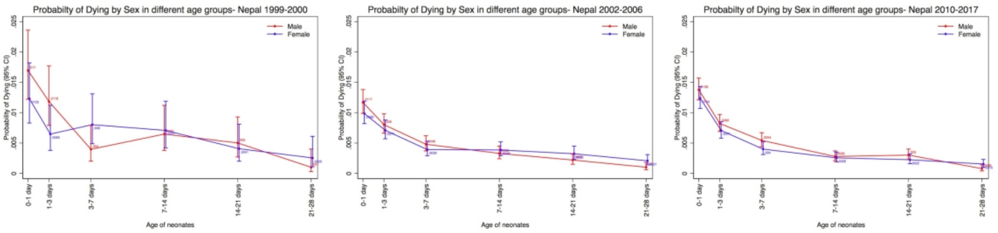

Figure 1: Sex difference in probability of dying for individual studies- NNIPS-3, CHX and NOMS (from left to right)

115x83mm (600 x 600 DPI)

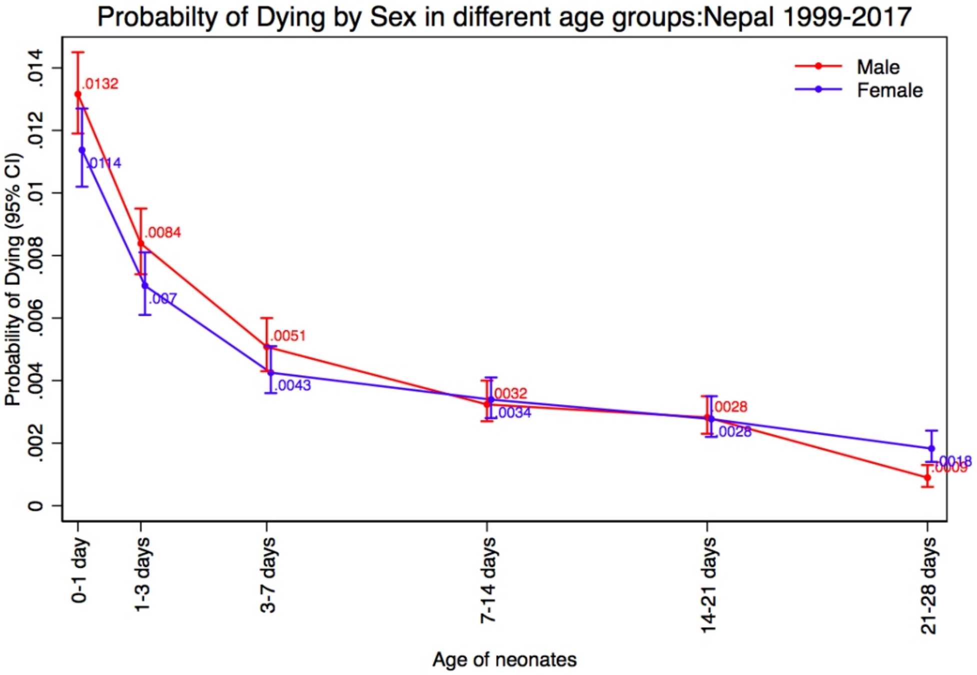

Figure 2: Sex difference in probability of dying for Pooled Analysis, Nepal 1999-2017

426x310mm (72 x 72 DPI)

ANNEX

Table 1: Mortality Rates by age and sex for NNIPS-3

NNIPS-3 (1999-2000) N=4,127										
	Males (N=2082)					Females(N=2045)				
**Age Group**	**Deaths**	**Person Year**	**Death Rate**	**Probability of Dying**	**Cumulative Probability (Mortality)**	**Deaths**	**Person Year**	**Death Rate**	**Probability of Dying**	**Cumulative Probability (Mortality)**
0-1day	35	2064	0.0169575	.017	.0168	25	2031.5	0.0123063	.0123	.0122
1-3 days	24	4055	0.0059186	.0118	.0284	13	4014.0	0.0032387	.0065	.0186
3-7 days	8	8063	0.0009922	.004	.0322	16	7973.0	0.0020068	.008	.0265
7-14 days	13	14051	0.0009252	.0065	.0385	14	13852.0	0.0010107	.0071	.0333
14-21 days	10	13964	0.0007161	.005	.0433	8	13782.0	0.0005805	.0041	.0372
21-28 days	2	13927	0.0001436	.001	.0442	5	13744.0	0.0003638	.0025	.0397
**Total**	**92**	**56123**				**81**	**55396.48**			

Table 2: Mortality Rates by age and sex for Chlorhexidine Study

CHX Study(2002-2006) N=23,644										
	Males (N= 12,188)					Females(N=11,456)				
**Age Group**	**Deaths**	**Person Year**	**Death Rate**	**Probability of Dying**	**Cumulative Probability (Mortality)**	**Deaths**	**Person Year**	**Death Rate**	**Probability of Dying**	**Cumulative Probability (Mortality)**
0-1day	141	12065	0.0116866	.0117	.0116	112	11362	0.0098573	.0099	.0098
1-3 days	96	23924	0.0040127	.008	.0195	80	22559	0.0035463	.0071	.0168
3-7 days	57	47541	0.0011990	.0048	.0242	44	44879	0.0009804	.0039	.0207
7-14 days	39	82881	0.0004706	.0033	.0274	43	78208	0.0005498	.0038	.0244
14-21 days	26	82648	0.0003146	.0022	.0296	36	77929	0.0004620	.0032	.0276
21-28 days	12	82477	0.0001455	.001	.0305	23	77685	0.0002961	.0021	.0296
**Total**	**371**	**331536**				**338**	**312622**			

For peer review only

Table 3: Mortality Rates by age and sex for NOMS

NOMS (2010-2017) N=31,958										
	Males (N= 16,533)					Females(N=15,425)				
**Age Group**	**Deaths**	**Person Year**	**Death Rate**	**Probability of Dying**	**Cumulative Probability (Mortality)**	**Deaths**	**Person Year**	**Death Rate**	**Probability of Dying**	**Cumulative Probability. (Mortality)**
0-1day	225	16343	0.0137674	.0138	.0137	189	15266	0.0123804	.0124	.0123
1-3 days	133	32377	0.0041079	.0082	.0217	107	30273	0.0035345	.0071	.0193
3-7 days	87	64045	0.0013584	.0054	.027	60	59997	0.0010000	.004	.0232
7-14 days	44	110578	0.0003979	.0028	.0298	38	103879	0.0003658	.0026	.0257
14-21 days	47	109206	0.0004304	.003	.0327	33	102730	0.0003212	.0022	.0279
21-28 days	12	106394	0.0001128	.0008	.0334	22	100177	0.0002196	.0015	.0294
**Total**	**548**	**438943**				**449**	**412322**			

Table 4: Mortality Rates by age and sex for Pooled Study

Pooled Nepal Datasets (1999-2017) N=59,729										
	Males (N=30803)					Females(N=28926)				
**Age Group**	**Deaths**	**Person Year**	**Death Rate**	**Probability of Dying**	**Cumulative Probability (Mortality)**	**Deaths**	**Person Year**	**Death Rate**	**Probability of Dying**	**Cumulative Probability (Mortality)**
0-1day	401	30472	0.0131596	.0132	.0131	326	28660	0.0113748	.0114	.0113
1-3 days	253	60356	0.0041918	.0084	.0213	200	56846	0.0035183	.007	.0182
3-7 days	152	119649	0.0012704	.0051	.0263	120	112849	0.0010634	.0043	.0224
7-14 days	96	207511	0.0004626	.0032	.0294	95	195939	0.0004848	.0034	.0257
14-21 days	83	205818	0.0004033	.0028	.0322	77	194441	0.0003960	.0028	.0284
21-28 days	26	202798	0.0001282	.0009	.033	50	191606	0.0002610	.0018	.0302
**Total**	**1011**	**826603**				**868**	**780340**			

For peer review only

STROBE Statement—checklist of items that should be included in reports of observational studies

Does higher early neonatal mortality in boys reverse over the neonatal period? A pooled analysis from three trials of Nepal

	Item No	Recommendation	Page No
**Title and abstract**	1	(*a*) Indicate the study's design with a commonly used term in the title or the abstract	1
		(*b*) Provide in the abstract an informative and balanced summary of what was done and what was found	2
**Introduction**			
Background/rationale	2	Explain the scientific background and rationale for the investigation being reported	3,4,5
Objectives	3	State specific objectives, including any prespecified hypotheses	5
**Methods**			
Study design	4	Present key elements of study design early in the paper	6
Setting	5	Describe the setting, locations, and relevant dates, including periods of recruitment, exposure, follow-up, and data collection	6
Participants	6	(*a*) *Cohort study*—Give the eligibility criteria, and the sources and methods of selection of participants. Describe methods of follow-up<br>*Case-control study*—Give the eligibility criteria, and the sources and methods of case ascertainment and control selection. Give the rationale for the choice of cases and controls<br>*Cross-sectional study*—Give the eligibility criteria, and the sources and methods of selection of participants	6,8
		(*b*) *Cohort study*—For matched studies, give matching criteria and number of exposed and unexposed<br>*Case-control study*—For matched studies, give matching criteria and the number of controls per case	NA
Variables	7	Clearly define all outcomes, exposures, predictors, potential confounders, and effect modifiers. Give diagnostic criteria, if applicable	6,7
Data sources/ measurement	8*	For each variable of interest, give sources of data and details of methods of assessment (measurement). Describe comparability of assessment methods if there is more than one group	6,7
Bias	9	Describe any efforts to address potential sources of bias	6,7
Study size	10	Explain how the study size was arrived at	8
Quantitative variables	11	Explain how quantitative variables were handled in the analyses. If applicable, describe which groupings were chosen and why	6,7
Statistical methods	12	(*a*) Describe all statistical methods, including those used to control for confounding	6,7
		(*b*) Describe any methods used to examine subgroups and interactions	7
		(*c*) Explain how missing data were addressed	9
		(*d*) *Cohort study*—If applicable, explain how loss to follow-up was addressed<br>*Case-control study*—If applicable, explain how matching of cases and controls was addressed<br>*Cross-sectional study*—If applicable, describe analytical methods taking account of sampling strategy	6
		(*e*) Describe any sensitivity analyses	

Continued on next page

For peer review only - http://bmjopen.bmj.com/site/about/guidelines.xhtml

For peer review only

**Results**			
Participants	13*	(a) Report numbers of individuals at each stage of study—eg numbers potentially eligible, examined for eligibility, confirmed eligible, included in the study, completing follow-up, and analysed	8
		(b) Give reasons for non-participation at each stage	NA
		(c) Consider use of a flow diagram	NA
Descriptive data	14*	(a) Give characteristics of study participants (eg demographic, clinical, social) and information on exposures and potential confounders	6
		(b) Indicate number of participants with missing data for each variable of interest	9
		(c) *Cohort study*—Summarise follow-up time (eg, average and total amount)	NA
Outcome data	15*	*Cohort study*—Report numbers of outcome events or summary measures over time	
		Case-control study—Report numbers in each exposure category, or summary measures of exposure	
		Cross-sectional study—Report numbers of outcome events or summary measures	9
Main results	16	(*a*) Give unadjusted estimates and, if applicable, confounder-adjusted estimates and their precision (eg, 95% confidence interval). Make clear which confounders were adjusted for and why they were included	6,9
		(*b*) Report category boundaries when continuous variables were categorized	6
		(*c*) If relevant, consider translating estimates of relative risk into absolute risk for a meaningful time period	NA
Other analyses	17	Report other analyses done—eg analyses of subgroups and interactions, and sensitivity analyses	7
**Discussion**			
Key results	18	Summarise key results with reference to study objectives	9
Limitations	19	Discuss limitations of the study, taking into account sources of potential bias or imprecision. Discuss both direction and magnitude of any potential bias	15
Interpretation	20	Give a cautious overall interpretation of results considering objectives, limitations, multiplicity of analyses, results from similar studies, and other relevant evidence	15
Generalisability	21	Discuss the generalisability (external validity) of the study results	15
**Other information**			
Funding	22	Give the source of funding and the role of the funders for the present study and, if applicable, for the original study on which the present article is based	16

*Give information separately for cases and controls in case-control studies and, if applicable, for exposed and unexposed groups in cohort and cross-sectional studies.

Note: An Explanation and Elaboration article discusses each checklist item and gives methodological background and published examples of transparent reporting. The STROBE checklist is best used in conjunction with this article (freely available on the Web sites of PLoS Medicine at http://www.plosmedicine.org/, Annals of Internal Medicine at http://www.annals.org/, and Epidemiology at http://www.epidem.com/). Information on the STROBE Initiative is available at www.strobe-statement.org.

For peer review only
